# Molecular signature of hypersaline adaptation: insights from genome and proteome composition of halophilic prokaryotes

**DOI:** 10.1186/gb-2008-9-4-r70

**Published:** 2008-04-09

**Authors:** Sandip Paul, Sumit K Bag, Sabyasachi Das, Eric T Harvill, Chitra Dutta

**Affiliations:** 1 Bioinformatics Center, Indian Institute of Chemical Biology, 4, Raja SC Mullick Road, Kolkata - 700 032, India; 2 Department of Biology, The Pennsylvania State University, Mueller Lab, University Park, PA 16802, USA; 3 Department of Veterinary and Biomedical Sciences, The Pennsylvania State University, University Park, PA 16802, USA; 4 Structural Biology and Bioinformatics Division, Indian Institute of Chemical Biology, 4, Raja SC Mullick Road, Kolkata - 700 032, India

## Abstract

**Background:**

Halophilic prokaryotes are adapted to thrive in extreme conditions of salinity. Identification and analysis of distinct macromolecular characteristics of halophiles provide insight into the factors responsible for their adaptation to high-salt environments. The current report presents an extensive and systematic comparative analysis of genome and proteome composition of halophilic and non-halophilic microorganisms, with a view to identify such macromolecular signatures of haloadaptation.

**Results:**

Comparative analysis of the genomes and proteomes of halophiles and non-halophiles reveals some common trends in halophiles that transcend the boundary of phylogenetic relationship and the genomic GC-content of the species. At the protein level, halophilic species are characterized by low hydrophobicity, over-representation of acidic residues, especially Asp, under-representation of Cys, lower propensities for helix formation and higher propensities for coil structure. At the DNA level, the dinucleotide abundance profiles of halophilic genomes bear some common characteristics, which are quite distinct from those of non-halophiles, and hence may be regarded as specific genomic signatures for salt-adaptation. The synonymous codon usage in halophiles also exhibits similar patterns regardless of their long-term evolutionary history.

**Conclusion:**

The generality of molecular signatures for environmental adaptation of extreme salt-loving organisms, demonstrated in the present study, advocates the convergent evolution of halophilic species towards specific genome and amino acid composition, irrespective of their varying GC-bias and widely disparate taxonomic positions. The adapted features of halophiles seem to be related to physical principles governing DNA and protein stability, in response to the extreme environmental conditions under which they thrive.

## Abstract 2

A comparative genomic and proteomic study of halophilic and non-halophilic prokaryotes identifies specific genomic and proteomic features typical of halophilic species that are independent from genomic GC-content and taxonomic position.

## Background

Halophiles are organisms adapted to thrive in extreme conditions of salinity. There is a wide range of halophilic microorganisms belonging to the domains Archaea and Bacteria. The intra-cellular machinery of these prokaryotes has evolved to function at very high salt concentrations [1-5]. A detailed understanding of the molecular mechanisms involved in the halophilic adaptation not only provides insight into the factors responsible for genomic and proteomic stability under high salt conditions, but also has importance for potential applications in the field of protein engineering [6,7].

The stable and unique native structure of a protein is a basic requirement for its proper functioning [8-11]. To understand molecular adaptation in hypersaline environments, it is important to address fundamental problems involving protein stabilization and solubility. An apparent way to achieve protein stability is to choose and arrange amino acid residues in their primary sequences in a specific or selective way. Several earlier works have revealed the elevated frequencies of negatively charged residues on protein surfaces as one of the most prominent features of halophilic organisms [1,4,12-16]. The higher usage of negatively charged amino acid residues leads to organization of a hydrated salt ion network at the surface of the protein [17] and formation of salt bridges with strategically positioned basic residues [18], regulating the stability of proteins. But an increase of acidic residues on protein surfaces is not the only possible adaptation to high salinity [13,19]. Earlier works have also pointed towards relatively low hydrophobicity as another adaptation to high salt environments [4,20]. Therefore, a clear and comprehensive picture of protein signatures for halophilic adaptation remains elusive.

Several studies have suggested that high genomic GC-content (well above 60%) is also a common feature of extreme halophiles, presumably to avoid UV induced thymidine dimer formation and possible accumulation of mutations [14,19]. The newly sequenced genome of the extreme halophilic organism *Haloquadratum walsbyi *is so far the only exception, with a remarkably low genomic GC-content of 47.9% [21]. At the codon usage level, a strong GC-bias was observed for *Halobacterium *sp. NRC1 [14], but not for *H. walsbyi *[21]. Thus, at the genomic level, the GC-bias is not a universal feature for adaptation to high salinity and other specific features of nucleotide selection may also be involved.

The current report presents an extensive and systematic analysis of the genome and proteome composition of halophilic organisms, along with a comparative study of non-halophiles, with a view to characterize the molecular signatures of halophilic adaptation. We consider 6 completely sequenced obligatory halophiles and compare them with 24 non-halophiles from various phyla of both Archaea and Bacteria with comparable GC-content to minimize the phylogenetic influence and the effect of mutational bias on their nucleotide/amino acid usage patterns. We examine the preferences, if any, in amino acid replacements from non-halophile to halophile orthologs in an attempt to understand which residues are instrumental for halophilic adaptation. Finally, we show how observed patterns of change in amino acid compositions in response to extreme conditions of the environment are related to physical principles that govern stability of proteins under such conditions. This study examines in detail the genome and proteome-wide adaptations to extreme environments, knowledge of which has important potential applications in various fields, including the engineering of industrial biomolecules.

## Results

### Clustering of halophiles by amino acid composition

**Figure 1:**
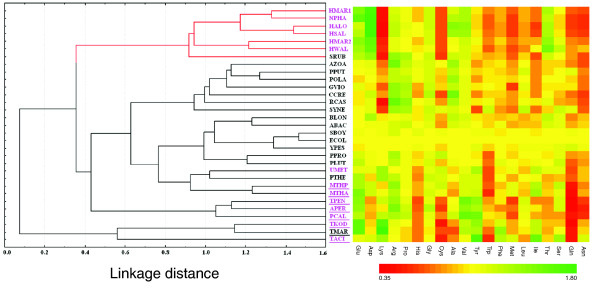
Grouping of halophiles and non-halophiles according to their standardized amino acid usage. Standardized amino acid composition of halophiles and non-halophiles grouped by unweighted pair group average clustering. The left panel depicts the unweighted pair group average clustering on the relative abundances of different amino acid residues in the encoded proteins of organisms with respect to those of *E. coli*. The distance in the clustering is Euclidean distance. The right panel is a pictorial representation of relative amino acid usage in the respective organisms. The over-representation or under-representation of amino acid residues in the organisms are shown in green and red colored blocks, respectively. Archaeal species are denoted in pink color and the species adapted to high temperature (optimum growth temperature ≥ 65°C) are underlined. Organism abbreviations are listed in Table 1.

Clustering on the relative abundances of different amino acid residues reveals a clear segregation of the halophilic organisms from the non-halophiles (Figure 1). The left panel of Figure 1 depicts the unweighted pair group average clustering on the relative abundances of different amino acid residues in the encoded proteins of the 6 extreme halophilic and 24 non-halophilic organisms under study (Table 1) with respect to those of *Escherichia coli*, while the right panel offers a pictorial representation of relative amino acid usage in the respective organisms. As the relative abundances of the residues increase from 0.35 to 1.80, the color of the respective block changes from red to green, that is, the greener the color, the more abundant is the residue in that organism compared to *E. coli*. Halophilic organisms show quite distinct usage of amino acid residues compared to non-halophiles, elucidated by the presence of either more red or green blocks in Figure 1. Among the prominent trends are significant increases in Asp, Glu, Val, and Thr residues and decreases in Lys, Met, Leu, Ile, and Cys in halophilic proteomes. Usage of Ile is lower in all halophiles except *H. walsbyi*, probably due to its significantly lower genomic GC-content (Table 1). The increase in negatively charged (Asp and Glu) and Thr residues and the decrease in Lys and strong hydrophobic residues (Ile, Met, Leu) are consistent with earlier reports [4,12,14,18,22]. A relatively higher frequency of Val in extreme halophiles compared to non-halophiles supports the observation of Madern *et al*. [15], but contradicts earlier propositions on under-representation of all strong hydrophobic residues in halophiles [4,23].

**Table 1:** General features of the 6 obligatory halophilic and 24 non-halophilic microbial genomes under study

Organism	Abbreviation	Group	ORFs under study	GC-content (%)
**Halophiles**				
*Haloarcula marismortui *ATCC 43049 Ch I	HMAR1	Euryarchaeota	2,705	62
*Haloarcula marismortui *ATCC 43049 Ch II	HMAR2	Euryarchaeota	217	57
*Halobacterium salinarum *DSM 671	HSAL	Euryarchaeota	2,191	68
*Halobacterium sp*. NRC-1	HALO	Euryarchaeota	1,782	67
*Haloquadratum walsbyi *DSM 16790	HWAL	Euryarchaeota	2,270	48
*Natronomonas pharaonis *DSM 2160	NPHA	Euryarchaeota	2,314	63
*Salinibacter ruber *DSM 13855	SRUB	Bacteroidetes/Chlorobi	2,631	66
				
**Non-halophiles**				
*Acidobacteria bacterium *Ellin345	ABAC	Acidobacteria	4,507	58
*Aeropyrum pernix *K1*	APER	Crenarchaeota	1,519	56
*Azoarcus sp*. EbN1	AZOA	Betaproteobacteria	3,673	65
*Bifidobacterium longum *NCC2705	BLON	Actinobacteria	1,643	60
*Caulobacter crescentus *CB15	CCRE	Alphaproteobacteria	3,453	67
*Escherichia coli *K12	ECOL	Gammaproteobacteria	3,829	50
*Gloeobacter violaceus *PCC 7421	GVIO	Cyanobacteria	3,947	62
*Methanosaeta thermophila *PT*	MTHP	Euryarchaeota	1,535	53
*Methanothermobacter thermautotrophicus *str. Delta H*	MTHA	Euryarchaeota	1,641	50
*Pelobacter propionicus *DSM 2379	PPRO	Deltaproteobacteria	3,404	58
*Pelodictyon luteolum *DSM 273	PLUT	Bacteroidetes/Chlorobi	1,926	57
*Pelotomaculum thermopropionicum *SI	PTHE	Firmicutes	2,544	53
*Polaromonas sp*. JS666	POLA	Betaproteobacteria	5,217	62
*Pseudomonas putida *KT2440	PPUT	Gammaproteobacteria	4,906	61
*Pyrobaculum calidifontis *JCM 11548*	PCAL	Crenarchaeota	1,932	57
*Roseiflexus castenholzii *DSM 13941	RCAS	Chloroflexi	4,077	61
*Shigella boydii *Sb227	SBOY	Gammaproteobacteria	3,660	47
*Synechococcus *sp. WH 7803	SYNE	Cyanobacteria	2,141	60
*Thermococcus kodakarensis *KOD1*	TKOD	Euryarchaeota	2,006	52
*Thermofilum pendens *Hrk 5*	TPEN	Crenarchaeota	1,647	58
*Thermoplasma acidophilum *DSM 1728*	TACI	Euryarchaeota	1,371	46
*Thermotoga maritima *MSB8*	TMAR	Thermotogae	1,695	46
*Uncultured methanogenic archaeon *RC-I	UMET	Euryarchaeota	2,800	55
*Yersinia pestis *Antiqua	YPES	Gammaproteobacteria	3,744	48

*Organisms with optimum growth temperature ≥ 65°C.

**Figure 2:**
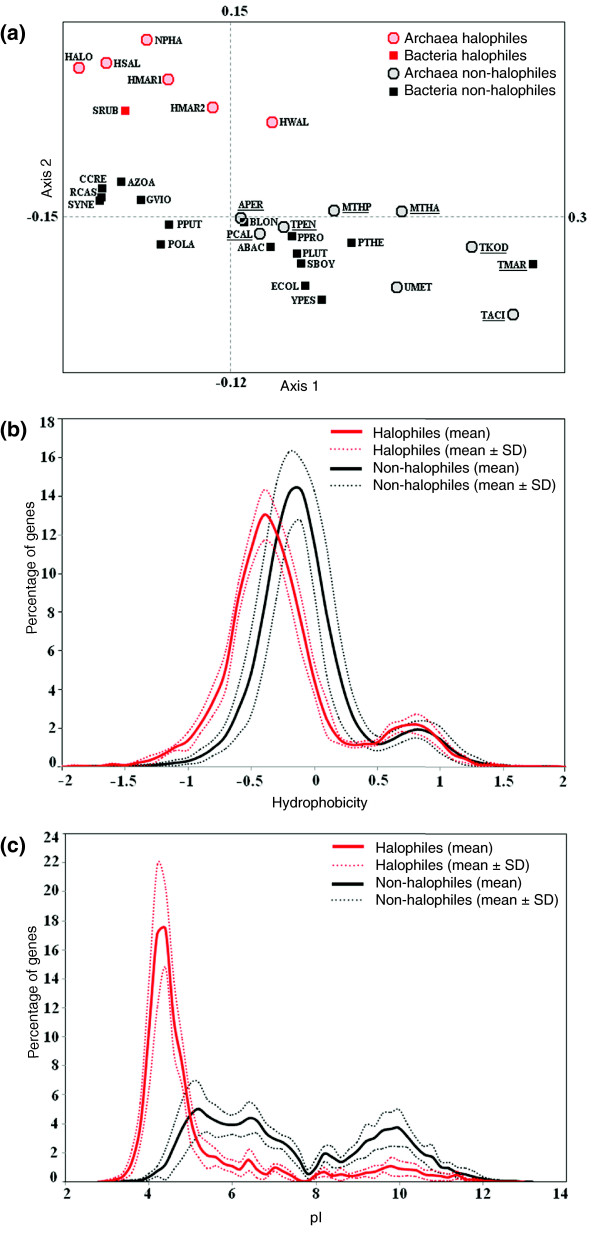
COA on amino acid usage and frequency distribution of genes on hydrophobicity and pI. **(a) **Positions of 24 non-halophiles and 6 halophiles on the plane defined by first and second major axes generated from COA on amino acid usage of encoded proteins. High temperature adapted organisms are underlined. **(b) **Distribution of genes on the basis of hydrophobicity of encoded proteins. **(c) **Distribution of genes on the basis of predicted pI of encoded proteins. Red and black color indicates halophiles and non-halophiles, respectively. Organism abbreviations are listed in Table 1.

Similar to the cluster analysis, correspondence analysis (COA) on amino acid usage also segregates the halophilic organisms from the non-halophiles along the second principal axis (Figure 2a). The first two principal axes of the COA contribute 16.29% and 13.79%, respectively, to the total variability. A strong negative correlation (r^2 ^= 0.57, *p *< 10^-7^) of axis 1 with the GC-content of the respective genomes identifies GC-bias as one of the major sources of inter-species variation in global amino acid composition, while the contributions to axis 2 come from hydrophobicity (negative correlation, with r^2 ^= 0.65, *p *< 10^-7^) and the ratio of negatively versus positively charged amino acid residues (positive correlation, with r^2 ^= 0.26, *p *< 10^-7^) of the encoded gene products of the organisms. This indicates, therefore, that the proteins of halophilic organisms are characterized by less hydrophobicity (or higher hydrophilicity) and relatively higher usage of negatively charged amino acids compared to non-halophile proteins. Figure 2b, c also supports the corollary that the features of halophilic proteomes are unique and quite distinct from those of non-halophiles with respect to hydrophobicity and usage of negatively charged amino acids (as predicted by isoelectric point distribution of encoded proteins).

All these trends are specifically exhibited by halophiles irrespective of their taxonomic origins and their genomic GC-content (Additional data file 1 and Table 1). For instance, five archaeal halophiles appear in a distinct cluster, far away from other closely related archaeal species like *Methanosaeta thermophila*, *Thermoplasma acidophilum *and so on (Figure 1). The salt-adapted bacteroidetes/chlorobi *Salinibacter ruber *also intermingles with these halophilic archaea - wide apart from *Pelodictyon luteolum*, its closest non-halophilic taxonomic relative. *H. walsbyi*, a halophile with relatively low GC-content (47.9%), appears in the same cluster along with the GC-rich halophiles, while the three non-halophilic species with similar GC-content and (*E. coli*, 50%; *Shigella boydii *47.4%; and *Yersinia pestis*, 47.8%) cluster with the other non-halophiles, most of which are characterized by much higher GC-content. It is worth mentioning at this point that organisms with high growth temperature also cluster together (Figure 1), of which two methanogenic organisms (*M. thermophila *and *Methanothermobacter thermautotrophicus*) share the same node. The distinct branching pattern of three thermophiles with relatively low genomic GC-content (*T. acidophilum*, *Thermotoga maritima *and *Thermococcus kodakarensis*) suggests that the overall GC-content also plays a significant role in shaping the amino acid composition of such organisms, as observed previously by Kreil and Ouzounis [24]. The exact topology of the cluster and values indicated by the colored blocks depend on the choice of standardization and the algorithm used for their construction, but the resulting grouping of the organisms in Figure 1 does not change significantly from that obtained using actual amino acid compositions of the respective organisms. These observations point towards convergent evolution of halophilic proteomes for specific amino acid composition, despite their varying GC-bias and widely disparate taxonomic positions.

### Comparison with non-halophilic orthologs

A comparison of orthologous proteins (cytosolic and membrane proteins separately) between halophilic and non-halophilic organisms was performed to identify the underlying factors for halophilic adaptation in more detail. Table 2 summarizes different proteomic properties of four sets of orthologous cytosolic proteins between halophiles and non-halophiles. In all cases, there is a significant increase in negatively charged, hydrophobic (Val) and borderline hydrophobic (Thr) residues and a decrease in positively charged, large hydrophobic and Cys residues (Table 2). Among negatively charged residues, the abundance of Asp (44% for set I, 65% for set II, 69% for set III and 55% for set IV) is higher than that of Glu (16% for set I, 43% for set II, 26% for set III and 34% for set IV). Similar trends were observed for the membrane proteins, although fairly large differences in amino acid usage were not found (data not shown).

**Table 2:** Differences between various indices of four sets of halophile proteins and their non-halophilic orthologs

	Mean							
	
	Set I (287 pairs of orthologous proteins)		Set II (104 pairs of orthologous proteins)		Set III (584 pairs of orthologous proteins)		Set IV (574 pairs of orthologous proteins)	
				
Indices	SRUB proteins	PLUT proteins	HMAR1 proteins	PPUT proteins	HMAR1 proteins	MTHP proteins	NPHA proteins	UMET proteins
Average hydrophobicity [52]	-0.37	-0.20*	-0.32	-0.12*	-0.37	-0.16*	-0.33	-0.20*
Positively charged residues (%)	10.33	12.26*	8.10	10.44*	8.84	13.03*	9.08	12.92*
Negatively charged residues (%)	17.13*	13.40	18.87*	12.32	19.54*	14.01	19.38*	13.50
Isoelectric point	5.09	6.61*	4.38	6.31*	4.46	6.70*	4.49	6.96*
Cysteine residue (%)	0.74	0.96^†^	0.85	1.23^†^	0.82	1.25*	0.81	1.18*
Valine residue (%)	8.07*	7.42	8.69^†^	7.95	8.84*	8.16	9.03*	8.01
Threonine residue (%)	5.84*	5.12	6.22*	5.09	6.08*	4.17	6.11*	5.29
Large, hydrophobic residues (I, L, M, F) (%)	18.62	22.06*	17.72	21.12*	16.79	22.26*	16.72	21.46*

*Significance at *p *< 10^-5 ^; ^†^significance at *p *< 10^-3^. Organism abbreviations are listed in Table 1.

We determined the frequencies of all possible amino acid replacements (that is, (20 × 19)/2 = 190 possible pairs of replacements) between the orthologous sequences in the direction from non-halophile to halophile proteins (Additional data files 2-5). There are 59 (31% of all possible pairs), 51 (26% of all possible pairs), 81 (42% of all possible pairs) and 76 (40% of all possible pairs) pairs of amino acids for sets I, II, III and IV, respectively, that have significant directional replacement bias (*p *< 10^-2 ^for set II; *p *< 10^-3 ^for set I; and *p *< 10^-6 ^for sets III and IV). They contribute 56%, 52%, 66% and 63% of the replacements for set I (28,267 of the 50,403 observed replacements), set II (10,815 of the 20,685 observed replacements), set III (69,974 of the 105,771 observed replacements) and set IV (68,243 of the 107,474 observed replacements), respectively (Additional data files 6-9). The top 20 replacements in all these sets suggest that there are two clear trends in amino acid substitution patterns in terms of highest gain as well as highest ratio (Table 3). These are: Lys (non-halophile) substituted by other residues (halophile); and other residues (non-halophile) substituted by acidic residues, especially Asp (halophile). Lys→Asp topped the list of most significantly biased substitutions in terms of ratio in all the sets under study, indicating that this trend is independent of GC-composition and phylogeny. Another notable trend is Ile/Leu (non-halophile) substituted by Val/other residues (halophile). In set II, where the orthologs are of similar GC-composition, there is a prevalence of overall gain in Asp, Glu, Val and Thr, which are also gained in sets I, III and IV in halophile from non-halophile orthologs (Table 3). Thus, there is a prevalence of overall gain in Asp, Glu, Val and Thr and the most prominent losses common in all four groups are Lys, Ile, Met, Leu and Cys in halophile from non-halophile orthologs. This result suggests that such gains and losses indeed represent an imprint of halophilic adaptation, and not the dragging effect of mutational bias or taxonomic differences.

**Table 3:** Top 20 amino acid pairs of 4 orthologous groups according to differences and ratios in number of forward (non-halophiles to halophiles) and backward (halophiles to non-halophiles) replacements

	Most biased in gain					Most biased in ratio				
		
	Pair	Ratio	Forward no.	Reverse no.	Gain	Pair	Ratio	Forward no.	Reverse no.	Gain
Set I (orthologous proteins	I→V	1.82	1,632	895	737	C→D	9	27	3	24*
of PLUT and SRUB)	I→L	1.85	1,195	647	548	I→D	8.5	34	4	30*
	K→E	4.09	704	172	532	I→P	8.43	59	7	52*
	K→R	2.06	856	416	440	K→D	6.35	438	69	369
	K→D	6.35	438	69	369	I→R	6.18	105	17	88
	E→D	1.39	1,214	874	340	L→D	4.46	107	24	83
	G→D	2.43	485	200	285	K→P	4.38	140	32	108
	S→A	1.53	818	534	284	K→E	4.09	704	172	532
	S→D	2.56	438	171	267	L→W	4.08	49	12	37
	N→D	2.45	431	176	255	M→P	3.43	48	14	34
	K→Q	2.84	330	116	214	M→E	3.28	95	29	66
	S→T	1.52	616	405	211	F→H	3.27	85	26	59
	L→V	1.31	833	635	198	I→E	3.03	94	31	63
	R→D	2.57	308	120	188	M→R	2.95	112	38	74
	S→E	1.82	403	221	182	L→E	2.91	201	69	132
	A→D	1.77	415	235	180	K→Q	2.84	330	116	214
	K→A	2.47	287	116	171	K→G	2.74	167	61	106
	L→R	2.65	252	95	157	L→R	2.65	252	95	157
	K→T	2.64	230	87	143	K→T	2.64	230	87	143
	R→E	1.39	497	357	140	R→D	2.57	308	120	188
										
Set II (orthologous proteins	K→E	5.19	306	59	247	K→D	8.56	214	25	189
of PPUT and HMAR1)	I→V	1.62	521	321	200	L→D	5.6	56	10	46
	L→V	1.75	462	264	198	K→E	5.19	306	59	247
	A→E	2.28	351	154	197	Q→D	5.11	189	37	152
	K→D	8.56	214	25	189	K→S	4.92	59	12	47
	A→D	2.93	264	90	174	H→D	4.44	80	18	62
	Q→E	2.44	266	109	157	I→D	4.2	21	5	16
	Q→D	5.11	189	37	152	I→Y	4	40	10	30
	G→D	2.41	210	87	123	P→H	3.8	19	5	14
	R→D	2.71	160	59	101	M→E	3.63	29	8	21
	R→E	1.95	207	106	101	L→P	3.43	48	14	34
	N→D	2.35	169	72	97	Y→D	3.38	27	8	19
	E→D	1.25	429	343	86	K→T	3.29	102	31	71
	S→T	1.46	249	170	79	C→A	3.24	81	25	56
	S→D*	1.9	152	80	72	L→E	3.13	100	32	68
	K→T	3.29	102	31	71	K→G	2.95	65	22	43
	A→T	1.55	198	128	70	A→D	2.93	264	90	174
	P→D	2.82	107	38	69	K→P	2.91	32	11	21
	L→I*	1.25	349	280	69	P→D	2.82	107	38	69
	L→E	3.13	100	32	68	I→E	2.75	44	16	28
										
Set III (orthologous proteins	I→V	2.23	3,368	1,513	1,855	C→Q	10.00	30	3	27*
of MTHP and HMAR1)	R→E	2.62	1,836	701	1,135	K→D	7.30	883	121	762
	R→D	4.34	1,306	301	1,005	M→D	7.28	182	25	158
	E→D	1.52	2,576	1,693	883	C→D	5.31	69	13	56
	K→E	4.11	1,162	283	879	I→Q	5.10	148	29	119
	I→L	1.70	2,087	1,230	857	M→Q	4.91	157	32	125
	S→D	2.84	1,312	462	850	C→E	4.86	68	14	54
	K→D	7.30	883	121	762	I→D	4.80	192	40	152
	L→V	1.60	1,978	1,237	741	L→D	4.49	337	75	262
	G→D	2.35	1,183	504	679	M→H	4.44	80	18	62
	S→A	1.61	1,556	967	589	K→Q	4.42	407	92	315
	S→E	2.00	1,054	527	527	R→D	4.34	1,306	301	1,005
	R→A	2.14	978	458	520	K→E	4.11	1,162	283	879
	R→T	3.10	740	239	501	K→G	4.04	331	82	249
	L→A	2.11	946	448	498	C→N	4.00	32	8	24*
	I→A	2.76	756	274	482	I→W	3.88	62	16	46
	I→T	3.84	572	149	423	I→T	3.84	572	149	423
	V→T	1.88	892	475	417	M→E	3.81	259	68	191
	N→D	2.23	753	337	416	K→T	3.79	425	112	313
	R→Q	2.45	697	284	413	W→D	3.69	48	13	35*
										
Set IV (orthologous proteins	I→V	2.58	3,584	1,389	2,195	K→D	12.93	1,461	113	1,348
of UMET and NPHA)	K→E	8.72	2,023	232	1,791	K→E	8.72	2,023	232	1,791
	K→D	12.93	1,461	113	1,348	K→A	8.28	993	120	873
	K→R	3.01	1,623	540	1,083	K→G	6.57	519	79	440
	I→L	1.95	2,135	1,096	1,039	M→D	6.50	130	20	110
	K→A	8.28	993	120	873	M→R	5.59	246	44	202
	N→D	3.20	1,024	320	704	K→T	5.44	685	126	559
	S→A	1.65	1,515	920	595	C→R	5.00	60	12	48
	K→T	5.44	685	126	559	K→P	4.92	300	61	239
	I→A	2.78	760	273	487	I→E	4.75	318	67	251
	M→L	1.93	1,000	517	483	I→H	4.48	103	23	80
	G→D	1.85	1,037	561	476	K→S	4.42	570	129	441
	S→D	1.98	915	462	453	I→D	4.05	174	43	131
	I→T	3.88	606	156	450	C→N	4.00	32	8	24*
	K→S	4.42	570	129	441	K→Q	3.95	510	129	381
	K→G	6.57	519	79	440	I→T	3.88	606	156	450
	V→A	1.41	1,464	1,040	424	M→H	3.82	65	17	48
	L→V	1.32	1,753	1,329	424	I→R	3.81	278	73	205
	R→E	1.57	1,136	724	412	M→E	3.48	212	61	151
	E→D	1.23	2,181	1,774	407	M→P	3.41	75	22	53

All the replacements of amino acid pairs are significant at *p *< 10^-3 ^for set I, *p *< 10^-2 ^for set II, and *p *< 10^-6 ^for sets III and IV, except replacements marked with asterisks. Organism abbreviations are listed in Table 1.

### Secondary structure comparison of orthologous sequences

**Figure 3:**
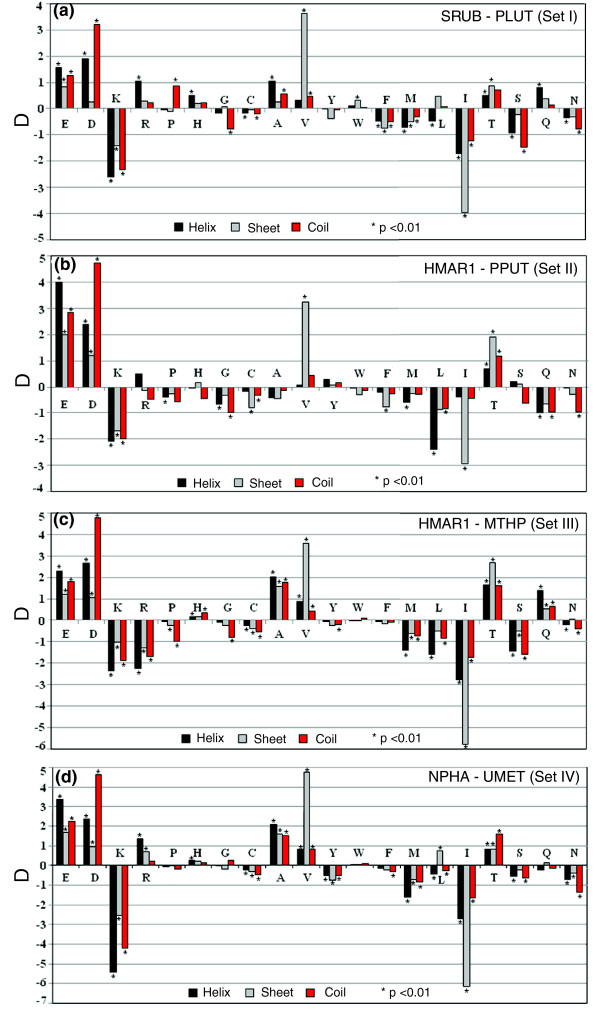
Variations in amino acid content of different secondary structural regions. The differences in the contributions of individual amino acids to secondary structural regions in orthologous proteins from **(a) ***S. ruber *and *P. luteolum *(set I), **(b) ***H. marismortui *chromosome I and *P. putida *(set II), **(c) ***H. marismortui *chromosome I and *M. thermophila *(set III) and **(d) ***N. pharaonis *and methanogenic archaeon (set IV). The differences were derived as D = (Frequency of halophile amino acid residue - Frequency of non-halophile amino acid residue). Black, grey and red bars indicate helix, sheet and coil regions, respectively, and asterisks indicates significant differences at *p *< 10^-2^. Organism abbreviations are listed in Table 1.

The results of various traits observed from predicted secondary structure for four sets of orthologs are shown in Table 4. For all sets there are higher propensities for the formation of random coil regions and lower propensities for the formation of helical structures in the encoded proteins of halophiles compared to non-halophile proteins. We measured all nine types of secondary structure replacements of amino acid residues between four sets of orthologous protein sequences from non-halophilic organisms to halophilic organisms (Table 5). In all data sets, residues having higher propensities for helix or sheet formation in non-halophile proteins are replaced by residues having higher propensities for coil formation in halophile orthologs. The differences in the contributions of individual amino acids to the predicted secondary structures between halophiles and non-halophiles for four sets of proteins are given in Figure 3. The large hydrophobic (Leu, Met) and positively charged (Lys) amino acids with higher helical propensity are significantly underrepresented, whereas the Asp residue, with higher coil forming propensity, is greatly over-represented in halophile proteins. There is also a significant decrease in Ile and an increase in Val and Thr residues, all of which have higher sheet-forming propensities.

**Table 4:** Secondary structure traits of residues of proteins of four sets of halophile proteins and their non-halophile orthologs

	Mean							
	
	Set I		Set II		Set III		Set IV	
				
Indices (%)	SRUB proteins	PLUT proteins	HMAR1 proteins	PPUT proteins	HMAR1 proteins	MTHP proteins	NPHA proteins	UMET proteins
Alpha helix	35.56	37.71*	31.02	37.08*	33.22	38.09*	34.11	36.91*
Beta sheet	14.06	15.42*	15.15	15.80	14.44	16.07*	14.91	16.34*
Coil	50.39*	46.87	53.83*	47.12	52.34*	45.84	50.98*	46.75

*Significance at *p *< 10^-5^. Organism abbreviations are listed in Table 1.

**Table 5:** Secondary structure replacements of four sets of halophile proteins and their non-halophile orthologs

		SRUB		
		
		Alpha helix	Beta sheet	Coil
PLUT	Alpha helix	1.000 (26212)	**0.997 **(2882)	**1.355 **(9976)
	Beta sheet	**1.003 **(2891)	1.000 (8352)	**1.26 **(5019)
	Coil	**0.866 **(7376)	**0.752 **(3974)	1.000 (35021)
				
		HMAR1		
		
		Alpha helix	Beta sheet	Coil
PPUT	Alpha helix	1.000 (8699)	**1.30 **(1260)	**1.59 **(4447)
	Beta sheet	**0.77 **(969)	1.000 (2959)	**1.342 **(2098)
	Coil	**0.629 **(2796)	**0.745 **(1563)	1.000 (12994)
				
		HMAR1		
		
		Alpha helix	Beta sheet	Coil
MTHP	Alpha helix	1.000 (41533)	**1.116 **(6115)	**1.451 **(21295)
	Beta sheet	**0.896 **(5480)	1.000 (12981)	**1.398 **(11012)
	Coil	**0.689 **(14671)	**0.715 **(7877)	1.000 (57559)
				
		NPHA		
		
		Alpha helix	Beta sheet	Coil
UMET	Alpha helix	1.000 (39245)	**0.850 **(5313)	**1.328 **(21724)
	Beta sheet	**1.176 **(6247)	1.000 (14685)	**1.303 **(10098)
	Coil	**0.753 **(16358)	**0.767 **(7747)	1.000 (59257)

Values represent ratios in number of forward (non-halophiles to halophiles) and backward (halophiles to non-halophiles) replacements. Entries in bold are significant at *p *< 10^-3^. Organism abbreviations are listed in Table 1.

### Comparison between known protein structures

**Figure 4:**
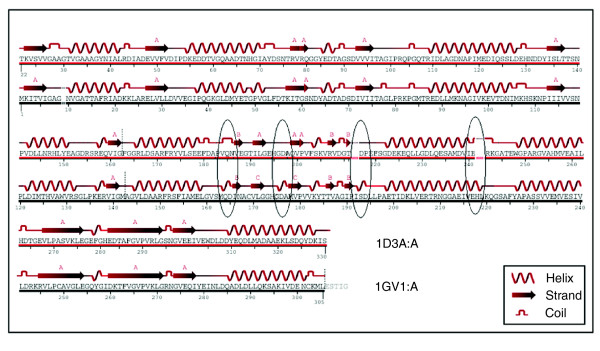
Secondary structural comparison. Comparison of secondary structured regions of crystal structures calculated by DSSP for aligned orthologous sequences of MDH proteins from *H. marismortui *(1D3A) and *C. vibrioforme *(1GV1). Changes in secondary structures in aligned regions of non-halophile (black line) and halophile (red line) protein are marked by ovals whereas gapped regions are marked by dotted lines.

One pair of crystal structures of the protein malate dehydrogenase (MDH) from halophilic *Haloarcula marismortui *and its ortholog from non-halophilic *Chlorobium vibrioforme *was selected and the secondary structures of these proteins were calculated with the help of the program MolMol. There is a marked decrease in helix forming regions in *H. marismortui *MDH (43.7% decrease) compared to *C. vibrioforme *MDH (48.5% decrease). The comparison of aligned sequences of secondary structure regions using the DSSP program also lends supports to this notion (Figure 4). In the MDH of *H. marismortui *(pI = 4.2; Hydrophobicity = -0.408), the cumulative frequency of Asp and Glu is 20.5%, whereas in *C. vibrioforme *MDH (pI = 5.3; Hydrophobicity = 0.136) it is 12.9%.

### Amino acid preference in halophiles is not a consequence of mono-nucleotide composition bias

**Figure 5:**
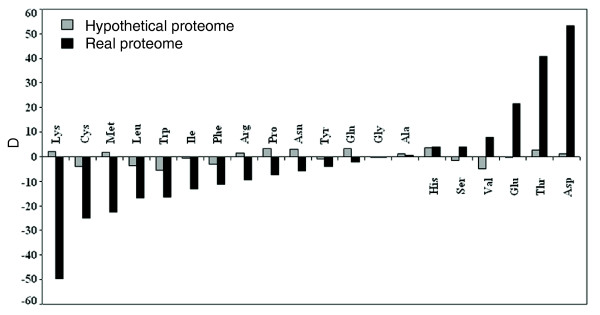
Average amino acid composition of real and hypothetical proteomes. Differences between average amino acid composition of real proteomes (black bars) and hypothetical proteomes simulated from reshuffled DNA (gray bars) of halophilic and non-halophilic organisms. The differences were derived as D = [(Avgerage halophilic/Avgerage nonhalophilic) -1] × 100.

The distinct amino acid usage pattern in halophiles might have originated from compositional bias operating at the nucleotide level, or from the preference for, or avoidance of, specific amino acid residues as a tool for halophilic adaptation. With a view to distinguish between these two possibilities, we randomly re-shuffled the nucleotides in the coding sequences of all genomes and calculated the average amino acid composition of the hypothetical protein sequences of halophiles and non-halophiles obtained from the theoretical translation of the reshuffled gene sequences. If the selection had operated at the mono-nucleotide level, proteins translated from such randomly reshuffled hypothetical sequences of halophiles should feature similar trends as depicted by their true proteomes, since the nucleotide bias of the reshuffled sequences would have remained the same as those of the real gene sequences. On the contrary, if the distinct amino acid composition of halophile proteomes had evolved due to environmental adaptation of these extremophiles, the trends in amino acid usage in reshuffled hypothetical sequences would differ from those of actual halophilic proteins. In Figure 5, the striking difference between average amino acid compositions of halophilic and non-halophilic organisms for real proteomes and hypothetical proteomes simulated from reshuffled DNA suggest that some factors, other than the mono-nucleotide usage, influence the amino acid composition of proteins to maintain structure and function under halophilic conditions.

### Genomic signature of halophiles

**Figure 6:**
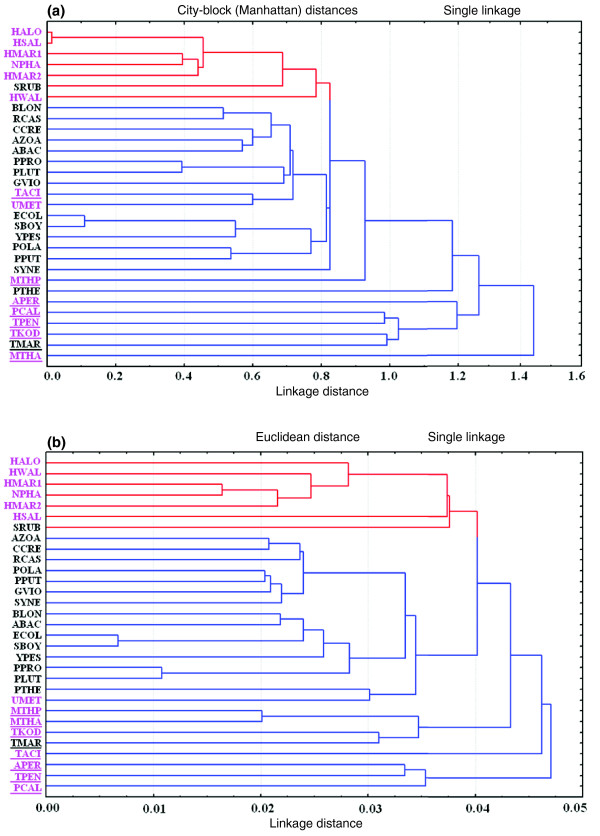
Clustering on dinucleotide values. **(a) **Clustering on dinucleotide abundance values of the genomes of all the organisms under study by city-block (Manhattan) distance. **(b) **Clustering made by dinucleotide frequencies at the first and second codon positions of genes for all organisms under study. The distance in the clustering is Euclidean distance. Red and blue lines signify halophilic and non-halophilic organisms, respectively. Archaeal species are written in pink and the species adapted to high temperature are underlined. Organism abbreviations are listed in Table 1.

We calculated the dinucleotide abundance of all genomes to find out whether any specific nucleotide composition has significant influence on the genomic signature of obligatory halophiles. Clustering on dinucleotide abundance by city-block (Manhattan) distance clearly segregates the halophilic organisms (with over-representation of GA/TC, CG and AC/GT dinucleotides) from the non-halophiles (Figure 6a; Additional data file 10) irrespective of their archaeal or bacterial origin. In other words, the dinucleotide abundance profiles of halophilic genomes bear some common characteristics, which are quite distinct from those of non-halophiles and, hence, these may be regarded as specific genomic signatures for salt-adaptation. Cluster analysis on dinucleotide frequencies at the first and second codon positions of genes for all organisms also yielded separate clusters for halophiles and non-halophiles (Figure 6b). The higher frequencies of occurrence of GA, AC and GT dinucleotides at the first and second codon positions (Additional data file 11) undoubtedly reflect the requirements for Asp, Glu, Thr and Val residues in halophile protein sequences. Therefore, halophiles have a specific genome signature at the dinucleotide level, and this trend seems to be linked to a specific amino acid composition of proteins for halophilic adaptation. The high temperature adapted organisms seem to cluster together according to their overall dinucleotide relative abundance value except *Thermoplasma acidophilum*. However, on the basis of dinucleotide frequencies at the first and second codon positions of genes, these organisms cluster together irrespective of any phylogenetic relationship. In order to figure out the possible impact of the relative abundance of specific dinucleotides on the mechanical properties of halophilic genomes, we calculated the likelihood of their sequences forming a Z-DNA structure, using ZHunt software [25]. We found that there is a significant correlation (r^2 ^= 0.54, *p *< 10^-4^) between the propensity of DNA to flip from the B-form to the Z-form per kilobase of genome and the relative abundance of the CG dinucleotide.

### Synonymous codon usage bias in halophiles

**Figure 7:**
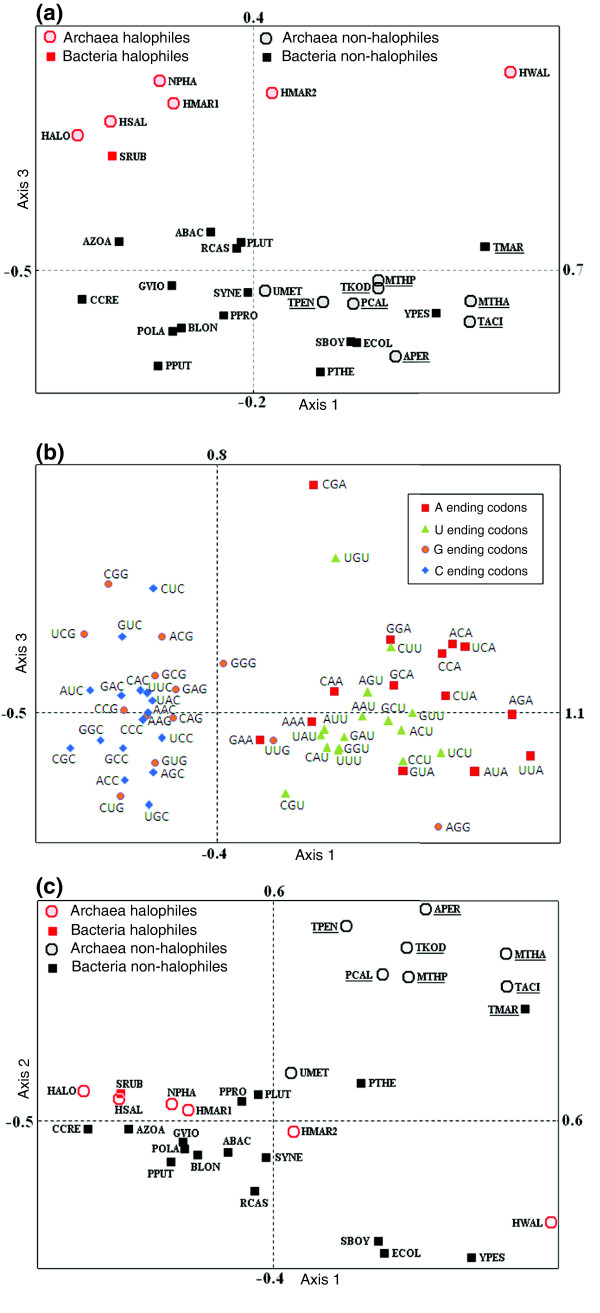
Correspondence analysis on RSCU. **(a) **Position of 24 non-halophiles and 6 halophiles along the first and third principal axes generated by COA on actual RSCU values of 82,927 predicted ORFs. High temperature adapted organisms are underlined. **(b) **Distribution of synonymous codons along the first and third principal axes of the COA on RSCU values of the genes of 82,927 predicted ORFs of 24 non-halophilic and 6 halophilic chromosomes. **(c) **Position of 24 non-halophiles and 6 halophiles along the first two principal axes generated by COA on actual RSCU values of 82,927 predicted ORFs. Species adapted to high temperature are underlined. Organism abbreviations are listed in Table 1.

In an attempt to examine whether the pattern of synonymous codon usage in halophiles follows any specific signature, COA was performed on the relative synonymous codon usage (RSCU) of 82,927 predicted open reading frames (ORFs) from 30 microbial genomes (listed in Table 1). The axis 1-axis 3 plot in Figure 7a of the COA on RSCU values exhibits two distinct clusters, the halophile and non-halophile genomes being segregated along the third major axis, whereas the axis 1-axis 2 plot in Figure 7c separates thermophilic organisms from mesophiles, indicating distinct usage of synonymous codons in thermophiles, as reported earlier [8,26]. This is the first report that the pattern of synonymous codon usage in the halophilic prokaryotes is different from that in the non-halophilic prokaryotes. Axis 1 values show highly significant correlation with the GC_3 _values (r^2 ^= 0.85, *p *< 10^-7^), indicating separation of genomes according to their genomic GC-content.

While differences in genomic GC-content and high temperature adaptation explain variations along the first and second major axes (representing 19.4 % and 11.1% of total variation, respectively) of the COA of RSCU, the variation along the third major axis (representing 9.1% of total variation) separates the halophiles from the non-halophiles. The distribution of codons along axis 3 (Figure 7b) depicts that the major contributors to this pattern are the distinct usage of synonymous codons encoding Arg (CGA and CGG being preferred by halophiles), Val (GUC is most preferred by halophiles), Thr (ACG is preferred by halophiles), Leu (CUC is the most preferred codon in halophiles) and Cys (UGU is generally preferred by halophiles). Comparison of codon usage values of 5,000 genes from both extremes of axis 3 shows that there are 18 and 14 codons, usage of which is significantly higher in the genes from the positive extreme and the negative extreme, respectively (Additional data file 12). Of the genes at the positive extreme, 97.5% are from halophiles, whereas 99.9% of the genes at the negative extreme are from non-halophiles. This means that in spite of their long-term evolutionary history, genes of the halophiles, in general, have converged to similar patterns of codon usage, which is quite distinct from the patterns followed by genes of non-halophilic organisms.

## Discussion

The present study discerns the nucleotide and amino acid biases in extreme halophiles and thereby characterizes the genomic/proteomic determinants of halophilic adaptation in prokaryotes. From this study, it appears that specific trends in amino acid usage are required for halophilic adaptation of organisms, irrespective of their genomic GC-content and taxonomic position. Evidence in favor of specific selection on dinucleotide and synonymous codon usage are apparent for halophiles (Figures 6a and 7a). Also, with regard to protein secondary structure, residues having lower propensities for forming alpha helical regions and higher propensities for forming coil-forming regions are preferred more in halophiles than non-halophiles (Table 4). All of these findings strongly support the notion of convergent evolution not only at the level of proteome composition, but also at the level of genome organization of the microorganisms adapted to high salt environments.

In order to subtract out the phylogenetic influence, we have included both bacterial and archaeal organisms in the dataset (Table 1). The dataset of halophilic organisms contains all available completely sequenced halophilic archaeal and bacterial species in the public domain, while the dataset of non-halophiles contains genome sequences of eight archaeal and sixteen bacterial species of diverse taxonomic origins. Among the archaeal non-halophiles are *M. thermophila *from Methanogens group II and *T. acidophilum *from Thermoplasmatales - organisms very close to haloarchaea as per the 16s rRNA tree (Additional data file 1). Among bacterial non-halophiles, we chose members from different bacterial phyla, such as proteobacteria, firmicutes, cyanobacteria, actinobacteria and especially *P. luteolum *from the bacteroidetes/chlorobi group to which the halophilic bacteria *S. ruber *belongs (Additional data file 1). It can be concluded, therefore, that the determinants of genomic/proteomic architecture in halophilic organisms are high salt adaptation specific, and transcend the boundary of phylogenetic relationships and the genomic GC-content of the species.

We have considered two chromosomes of *H. marismortui *in our analysis and found that the amino acid usage, dinucleotide relative abundance and synonymous codon usage of chromosome II are quite different from those of chromosome I (Figures 1, 2a, 6, and 7a), whereas they are relatively closer to each other in the 16s rRNA tree (Additional data file 1). This observation supports the earlier notion [27] that almost the entire chromosome II of *H. marismortui *might have been acquired later during evolution, while its rRNA operon might have originated through duplication and subsequent divergence from the rRNA operons of chromosome I.

Our study clearly indicates that halophile proteins prefer to use Asp, Glu, Val and Thr at the expense of Lys, Met, Leu, Ile and Cys. Among the residues favored in the halophilic proteome, Asp and Glu are negatively charged and may localize in patches on protein surfaces. By binding a network of hydrated cations, they help in the maintenance of protein activities at high salt concentrations [12,14,18,22]. The less common residues in high salt-adapted organisms include the positively charged residue Lys and several large and strongly hydrophobic residues like Leu, Ile and Met. An empirical correlation between halophilic adaptations of some proteins and their relatively low hydrophobicity was reported earlier [28]. It is interesting to note that although halophilic proteomes are, in general, characterized by lower hydrophobicity compared to non-halophiles, the usages of Val and Thr are significantly higher in them (Figure 1, Table 2). Usage of the strong hydrophobic residue Ile is also relatively higher in *H. walsbyi*, possibly due to its significantly lower genomic GC-content. At high salt concentrations, proteins are, in general, destabilized [29]. Halophile proteins have, therefore, evolved specific mechanisms that allow them to be both stable and soluble in the high cytoplasmic NaCl/KCl concentration. In this environment the hydrophobic residues of newly synthesized proteins are exposed to high salt concentrations, leading to non-specific inter- or intramolecular interactions of their side chains, which may compete with proper intramolecular burial within the correct conformation [30]. Probably to minimize this possibility, all soluble halophilic proteins have a lower number of hydrophobic residues. The increase in negative charge on the surface of halophile proteins counteracts the lower dielectric constant at high salinity and thus provides for enhanced protein solubility.

Our results show that there is a marked, significant difference in the predicted secondary structures of halophile and non-halophile proteins. In proteins with higher percentages of helix structure, there is an increased overall packing that imparts more rigidity [31] and, hence, a decrease in regions with helix-forming propensities in halophile proteins probably makes them more flexible. As protein flexibility and protein function are strongly linked [32,33], a reduction in helix-forming regions or, in other words, an enhancement in protein flexibility, might be a strategy of halophile proteins for adaptation to extreme salt environments. It is worth mentioning in this context that Radivojac *et al*. [34] divided native proteins into four flexibility categories and found that flexible but ordered proteins are characterized by higher average hydrophilicity and higher occurrence of negatively charged residues, especially Asp. From a structural viewpoint, Asp is recognized as an alpha-helix breaker, whereas Glu is favorable for alpha-helix formation [35]. The coiled regions of proteins are known to prefer Asp over Glu in general [36]. These could be plausible reasons why halophile proteins use Asp residues more than Glu residues.

A striking observation that has not been reported earlier and deserves mention is the consistent lower usage of Cys residues in halophile proteins. Cys residues are usually overrepresented in non-flexible regions due to the formation of rigid disulfide-bridges [33,37]. Avoidance of Cys residues in halophile gene products might give them more flexibility in high salt environments. Increased usage of Val in halophile proteins may also make them more flexible, because the strong hydrophobic residue Val has a lower helix formation propensity than other strong hydrophobic residues such as Leu, Met and Ile [36]. Thus, halophile proteins might have evolved to be more flexible but ordered and exhibit distinct secondary structure composition that has helped them to avoid aggregation and/or loss of function in extreme salt environments.

Like proteome composition, halophilic adaptation is also associated with a specific genome signature. The obligatory halophiles generally contain GC-rich genomes (well above 60%), except for *H. walsbyi *(genomic GC-content of 48.7%). A high GC-content in halophilic genomes is thought to help in avoiding UV-induced thymidine dimer formation and the possible accumulation of mutations in their specialized habitat (shallow coastal lagoons), characterized by high levels of UV irradiation [14,19]. In *H. walsbyi*, the disadvantage of a low GC-genome is thought to be partly compensated for by the presence of a relatively higher number (four copies) of photolyases [21]. Our analysis reveals that the genomes of all obligatory halophiles show definite dinucleotide abundance (higher abundance values for CG, GA/TC and AC/GT) compared to non-halophiles (Figure 6a, Additional file 10). The genomic signature revealed by dinucleotide abundance analysis for halophiles, in general, is not species-specific, but salt adaptation specific, and hence may be an outcome of convergent evolution. The higher frequency of GA, AC and GT dinucleotides at the first and second codon positions undoubtedly reflects the requirement for Asp, Glu, Thr and Val residues in halophile protein sequences. The higher occurrence of the CG dinucleotide leads to a higher stacking energy, thus imparting stability to genomic DNA [38]. It is also known that high salt concentrations have a strong influence on the transition of B-DNA to Z-DNA and the relative stabilization of Z-DNA increases with increasing salt concentration [39]. Hence, the enhancement of the total stacking interaction (base-pair stacking and deoxyribose purine stacking) could contribute to the propensity of short d(CG)n sequences to adopt the Z-conformation [40]. A significant correlation (r^2 ^= 0.54, *p *< 10^-4^) between the propensity of Z-DNA formation per kilobase in genomes with a relatively high abundance of CG dinucleotides supports this notion. We also observed that the pattern of synonymous codon usage in halophiles is significantly different from that in non-halophiles (Figure 7, Additional data file 12). Essentially, our results show that codon usage pattern among the 30 genomes (6 halophiles and 24 non-halophiles) is determined by three major factors: overall GC-bias (explained by the first major axis); a temperature dependent factor (explained by the second major axis); and a salinity dependent factor (explained by the third major axis). The COA on RSCU thus provides convincing evidence that synonymous codon usage in halophiles follows a similar trend, which is quite distinct from the trends observed in non-halophiles. Since the difference in synonymous codon usage between halophiles and non-halophiles is not due to a simple difference in the nucleotide content of the genomes, it seems that natural selection may be linked to the codon usage pattern of halophilic prokaryotes.

## Conclusion

The present study demonstrates the generality of the mechanisms of macromolecular adaptation of extreme salt-loving organisms, irrespective of their genomic GC-content and taxonomic position. At the protein level, these include: convergent evolution towards a specific proteome composition, characterized by low hydrophobicity; over-representation of acidic residues, especially Asp; higher usage of Val and Thr; lower usage of Cys; and a lower propensity for helix formation and a higher propensity for coil structure. Among the signatures of halophilic adaptation at the DNA level, the abundance of GA, AC and GT dinucleotides may partly be coupled with the specific amino acid requirements, while CG dinucleotide abundance may be an additional halophilic signature of DNA stability at high salt concentration. The synonymous codon usage in halophiles also seems to have converged to a single pattern regardless of their long-term evolutionary history.

## Materials and methods

### Sequence retrieval

All protein coding sequences of the chromosomes of 6 extreme halophiles (grow optimally in approximately 3.5 M NaCl) and 24 non-halophiles from Archaea (both euryarchaeota and crenarchaeota) and bacteria (including proteobacteria, firmicutes, cyanobacteria, actinobacteria, bacteroidetes/chlorobi, and so on) were retrieved from NCBI GenBank (version 145.0) and Halolex databases [41] (listed in Table 1). Except for *H. walsbyi*, all the extreme halophilic organisms are GC-rich, so to minimize the GC-compositional effect on amino acid usage comparison (as well as on codon usage), most of the chosen non-halophilic organisms are similarly GC-rich, while some others have GC-content comparable to that of *H. walsbyi*.

### Cluster analysis and correspondence analysis on amino acid usage

To find the differences in amino acid usage between extreme halophilic and non-halophilic organisms, the cluster analysis on amino acid composition was carried out using STATISTICA (version 6.0, published by Statsoft Inc., Tulsa, Oklahoma, USA) for all 30 organisms (Table 1). The amino acid usage of *E. coli *was chosen as a well-defined reference for standardization. Subsequently, using a program developed in Visual Basic, a 31 × 20 matrix was generated, where the rows and the columns correspond to data sources (that is, organisms in the cluster) and standardized amino acid usage values, respectively. The over-representation or under-representation of standardized amino acid usage values of the organisms in the matrix are shown in green or red colored blocks in Figure 1, respectively.

COA on amino acid usage was performed using the program CODONW 1.4.2 [42] to identify the major factors influencing the variation in amino acid frequencies. These analyses generate a series of orthogonal axes to identify trends that explain the variation within a dataset, with each subsequent axis explaining a decreasing amount of the variation.

### Dinucleotide analysis and reshuffling of DNA sequences

In order to identify any halophile-specific genome signature, dinucleotide abundance values [38,43] of genomes of halophiles and non-halophiles were calculated. Clustering of organisms on dinucleotide abundance values was done by the single linkage method and the nearest neighbor analysis was carried out using city-block (Manhattan) distance, calculated by summing the (absolute) differences between point coordinates. Dinucleotide frequencies at all three codon positions of each gene were also calculated and clustering was done using the single linkage method with Euclidean distance, which corresponds to the length of the shortest path between two points. Reshuffling of DNA sequences of ORFs was performed by swapping two randomly chosen nucleotides [44] in the sequence except start and stop codons (we rejected shuffling in cases where stop codons appeared within the ORFs), and repeating this swapping procedure for 3N times, where N is the length of the sequence.

### Amino acid exchange bias and secondary structure prediction with orthologous sequences

Four sets of orthologous sequences between halophiles and non-halophiles were identified (according to the comparable GC-content of the species and also according to the close phylogenetic relationships) using the BlastP program [45] using a cutoff of E = 1.0 × 10^-10^. Hits less than 60% similar and having more than 20% difference in length with the query were removed from the dataset. Putative membrane proteins and proteins likely to be secreted or localized to the cell surface, predicted using TMHMM2.0 [46] and SignalP3.0 [47], were also separated out. Using these criteria we identified four sets of orthologs. Set I included 287 orthologous proteins of two closely related species - the halophile *S. ruber *and the non-halophile *P. luteolum *- both belonging to the phylum bacteroidetes/chlorobi. Set II contained 104 orthologous sequences from two species with similar GC-content (Table 1) - the halophilic archaeon *H. marismortui *(Ch-I) and non-halophilic bacteria *Pseudomonas putida*. Set III contained 584 orthologous proteins from a halophilic and a thermophilic archaeon, namely *H. marismortui *(Ch-I) and *M. thermophila*. Set IV incorporated 574 orthologous proteins of the halophilic archaeon *Natronomonas pharaonis *and uncultured methanogenic archaeon RC -I.

The amino acid sequences of these four sets of orthologous genes were aligned using ClustalW [48] and the amino acid replacements were arranged in a 20 × 20 matrix using Substitution Pattern Analysis Software Tool (SPAST), a program in C++, developed in-house [49]. Secondary structure prediction of orthologous protein sequences were carried out using the Predator program [50]. The content of amino acid residues in helix, sheet and random coil regions were computed. Secondary structure replacements were calculated by aligning orthologous protein sequences. All these calculations were performed by a C++ program developed in-house.

While examining the trends in amino acid or secondary structure replacements, the direction of conversion of non-halophile proteins to extreme halophile proteins were taken by convention as the 'forward' direction. Under unbiased conditions, the ratio of forward to reverse replacements was expected to be 1:1 for each pair of replacements. To test this hypothesis, the observed and expected numbers (based on a 1:1 ratio) were recorded for each pair of residues belonging to a particular group. In all cases, the chi-square test was applied to assess the significance of the directional bias, if any, at significance levels of 10^-3 ^to 10^-6^. For each pair of replacements, the first and second rows of the 2 × 2 contingency table represented the number of replacements from one particular residue (say, i) to another (say, j) of the pair and the total count of the remaining replacements (say, k) from the residue i (where k ≠ j), respectively.

### Indices used to identify the trends in codon and amino acid usage

Indices like RSCU [51], GC-content at third codon position, amino acid frequencies and average hydrophobicity (Gravy score) [52] of protein coding sequences were calculated to find out the factors influencing codon and amino acid usage. The isoelectric point (pI) of each protein was calculated using the Expasy proteomics server [53]. Calculation of the likelihood of a DNA sequence forming a Z-DNA structure was done using the ZHunt server [25].

### Comparison with known protein secondary structures

We obtained one pair of protein structures for extreme halophilic and non-halophilic organisms from the Protein Data Bank. The pair contains (Blast *p*-value 1e^-38^) MDHs from *H. marismortui *(1D3A) [54] and *C. vibrioforme *(1GV1) [55]. The secondary structures of the modeled proteins were calculated using MolMol [56] and DSSP [57].

## Abbreviations

COA, correspondence analysis; MDH, malate dehydrogenase; ORF, open reading frame; RSCU, relative synonymous codon usage.

## Authors' contributions

SP and SKB made substantial contributions to the conception of the study, devised the overall strategy, performed genome sequence analysis and drafted the manuscript, developed relevant programs for sequence analysis and performed sequence alignment. SD participated in the initial work, development of the work plan and manuscript preparation. ETH made thoughtful and constructive suggestions during preparation of the manuscript. CD participated in the design and coordination of the study and revised the manuscript critically for important intellectual content. All authors read and approved the final manuscript.

## Additional data files

The following additional data are available with the online version of this paper. Additional data file 1 is a figure demonstrating the phylogenetic relationship with 16s rRNA. Additional data file 2 is a table listing the trends in amino acid replacements in *P. luteolum *and *S. ruber *orthologs. Additional data file 3 is a table listing the trends in amino acid replacements in *P. putida *and halophilic *H. marismortui *chromosome I orthologs. Additional data file 4 is a table listing the trends in amino acid replacements in *M. thermophila *and halophilic *H. marismortui *chromosome I orthologs. Additional data file 5 is a table listing the trends in amino acid replacements in *Methanogenic archaeon *and halophilic *N. pharaonis *orthologs. Additional data file 6 is a table listing the number of amino acid replacement from *P. luteolum *to halophilic *S. ruber *orthologs. Additional data file 7 is a table listing the number of amino acid replacements from *P. putida *to halophilic *H. marismortui *chromosome I orthologs. Additional data file 8 is a table listing the number of amino acid replacement from *M. thermophila *to halophilic *H. marismortui *chromosome I orthologs. Additional data file 9 is a table listing the number of amino acid replacement from *M. archaeon to *halophilic *N. pharaonis *orthologs. Additional data file 10 is a table listing the dinucleotide relative abundance of all the organisms under study. Additional data file 11 is a table listing the dinucleotide frequency at the first and second codon positions. Additional data file 12 is a table listing the RSCU values of positive and negative extreme genes.

## Supplementary Material

Additional data file 1 Phylogenetic relationship with 16s rRNA using the neighbor joining method with Tamura-Nei model. Click here for file

Additional data file 2 Trends in amino acid replacements in non-halophilic *P. luteolum *and halophilic *S. ruber *orthologs. Click here for file

Additional data file 3 Trends in amino acid replacements in non-halophilic *P. putida *and halophilic *H. marismortui *chromosome I orthologs. Click here for file

Additional data file 4 Trends in amino acid replacements in non-halophilic *M. thermophila *and halophilic *H. marismortui *chromosome I orthologs. Click here for file

Additional data file 5 Trends in amino acid replacements in non-halophilic *M. archaeon *and halophilic *N. pharaonis *orthologs. Click here for file

Additional data file 6 Number of amino acid replacements from non-halophilic *P. luteolum *to halophilic *S. ruber *orthologs. Click here for file

Additional data file 7 Number of amino acid replacements from non-halophilic *P. putida *to halophilic *H. marismortui *chromosome I orthologs. Click here for file

Additional data file 8 Number of amino acid replacements from non-halophilic *M. thermophila *to halophilic *H. marismortui *chromosome I orthologs. Click here for file

Additional data file 9 Number of amino acid replacements from non-halophilic *M. archaeon to *halophilic *N. pharaonis *orthologs. Click here for file

Additional data file 10 Dinucleotide relative abundance for all the organisms under study. Click here for file

Additional data file 11 Dinucleotide frequency at the first and second codon positions for all the organisms under study. Click here for file

Additional data file 12 RSCU of genes at the positive and negative extremes (5,000 each) of axis 3 of COA on RSCU. Click here for file
