# Supplementary material for: Molecular signature of hypersaline adaptation: insights from genome and proteome composition of halophilic prokaryotes
Source: Genome Biol. 2008 Apr 9;9(4):R70. doi: 10.1186/gb-2008-9-4-r70 (PMC2643941; doi:10.1186/gb-2008-9-4-r70)
Supplement: Additional data file 2 — Trends in amino acid replacements in non-halophilic P. luteolum and halophilic S. ruber orthologs. [file gb-2008-9-4-r70-S2.doc]

**Additional Data File 2:** Amino acid replacement matrix for *S. ruber* proteins and their *P. luteolum* orthologs (Set I)

|  | SRUB (halophile) | | | | | | | | | | | | | | | | | | | | |
| --- | --- | --- | --- | --- | --- | --- | --- | --- | --- | --- | --- | --- | --- | --- | --- | --- | --- | --- | --- | --- | --- |
| PLUT (non-halophile) |  | T | S | D | E | N | Q | R | K | H | W | Y | F | C | M | I | L | V | A | P | G |
|  | G | 1.11 | 0.79 | **2.42** | **1.57** | 0.91 | 0.94 | 0.94 | **0.37** | 0.93 | 0.8 | 0.94 | 1.00 | 0.51 | 0.79 | 0.37 | 0.55 | 0.72 | 0.87 | 0.94 | 1.00 |
|  | P | 0.71 | 0.77 | **2.26** | 0.97 | 0.71 | 0.63 | 0.68 | **0.23** | 0.47 | 0.33 | 0.63 | 0.47 | 0.25 | **0.29** | **0.12** | **0.51** | **0.49** | 0.78 | 1.00 |  |
|  | A | 1.06 | **0.65** | **1.77** | 1.09 | 0.89 | 1.04 | 1.06 | **0.40** | 1.02 | 1.00 | 0.75 | **0.44** | 0.76 | 0.69 | **0.50** | **0.71** | 0.85 | 1.00 |  |  |
|  | V | 1.16 | 0.65 | **1.85** | **1.94** | 1.46 | 1.45 | **1.43** | 0.90 | 0.88 | 2.00 | 0.66 | **0.49** | 0.77 | 0.71 | **0.55** | **0.76** | 1.00 |  |  |  |
|  | L | **1.66** | 0.81 | **4.46** | **2.91** | 0.69 | **2.25** | **2.65** | 1.08 | 1.67 | **4.08** | 0.93 | 0.80 | 1.11 | **0.75** | **0.54** | 1.00 |  |  |  |  |
|  | I | **2.55** | 1.42 | **8.50** | **3.03** | 1.33 | 2.60 | **6.18** | 1.26 | 3.75 | 2.25 | 1.47 | 1.19 | 1.93 | 1.31 | 1.00 |  |  |  |  |  |
|  | M | **2.10** | 1.32 | 2.60 | **3.28** | 1.38 | 1.88 | **2.95** | 1.66 | 1.80 | 2.86 | 1.15 | 1.04 | 1.86 | 1.00 |  |  |  |  |  |  |
|  | C | 1.96 | 1.47 | **9.00** | 4.50 | 1.20 | 2.25 | 2.18 | 1.25 | 2.25 | 1.33 | 4.40 | 1.07 | 1.00 |  |  |  |  |  |  |  |
|  | F | 1.57 | 1.23 | 2.43 | 1.70 | 0.42 | 3.10 | 2.55 | 1.06 | **3.27** | 1.53 | 1.48 | 1.00 |  |  |  |  |  |  |  |  |
|  | Y | 1.96 | 0.66 | 1.61 | 1.35 | 1.21 | 2.71 | 0.98 | 0.50 | **2.21** | 1.28 | 1.00 |  |  |  |  |  |  |  |  |  |
|  | W | 0.40 | 1.25 | 0.33 | 1.75 | 0.17 | 0.62 | 0.94 | 0.89 | 1.36 | 1.00 |  |  |  |  |  |  |  |  |  |  |
|  | H | 1.12 | **0.48** | **1.65** | 1.02 | **0.50** | 0.90 | 0.96 | **0.48** | 1.00 |  |  |  |  |  |  |  |  |  |  |  |
|  | K | **2.64** | 1.70 | **6.35** | **4.09** | 1.82 | **2.84** | **2.06** | 1.00 |  |  |  |  |  |  |  |  |  |  |  |  |
|  | R | 1.05 | **0.65** | **2.57** | **1.39** | 0.90 | **1.40** | 1.00 |  |  |  |  |  |  |  |  |  |  |  |  |  |
|  | Q | 1.03 | 0.72 | **1.92** | 1.09 | 0.55 | 1.00 |  |  |  |  |  |  |  |  |  |  |  |  |  |  |
|  | N | 1.55 | 0.96 | **2.45** | **1.84** | 1.00 |  |  |  |  |  |  |  |  |  |  |  |  |  |  |  |
|  | E | 0.81 | **0.55** | **1.39** | 1.00 |  |  |  |  |  |  |  |  |  |  |  |  |  |  |  |  |
|  | D | 0.81 | **0.39** | 1.00 |  |  |  |  |  |  |  |  |  |  |  |  |  |  |  |  |  |
|  | S | **1.52** | 1.00 |  |  |  |  |  |  |  |  |  |  |  |  |  |  |  |  |  |  |
|  | T | 1.00 |  |  |  |  |  |  |  |  |  |  |  |  |  |  |  |  |  |  |  |

Each element R_ij_ in the matrix represents the ratio of number of replacements of the residue i by the residue j in the forward direction (non-halophiles→halophiles) to that in the reverse direction. This means that if R_ij_ >1, the number of replacement (i)_non-halophiles_→(j)_Halophiles_  is higher than the number of replacement (j) _non-halophiles_ →(i) _Halophiles_ and if R_ij_ <1, the reverse is true. Bold ratios signifies the directional bias at p<10^-3^ respectively.
