# Supplementary material for: Molecular signature of hypersaline adaptation: insights from genome and proteome composition of halophilic prokaryotes
Source: Genome Biol. 2008 Apr 9;9(4):R70. doi: 10.1186/gb-2008-9-4-r70 (PMC2643941; doi:10.1186/gb-2008-9-4-r70)
Supplement: Additional data file 3 — Trends in amino acid replacements in non-halophilic P. putida and halophilic H. marismortui chromosome I orthologs. [file gb-2008-9-4-r70-S3.doc]

**Additional Data File 3:** Amino acid replacement matrix for *H. marismortui* (ch-I) and their *P. putida* orthologs (Set II)

|  | HMAR1 (halophile) | | | | | | | | | | | | | | | | | | | | |
| --- | --- | --- | --- | --- | --- | --- | --- | --- | --- | --- | --- | --- | --- | --- | --- | --- | --- | --- | --- | --- | --- |
| PPUT (non-halophile) |  | T | S | D | E | N | Q | R | K | H | W | Y | F | C | M | I | L | V | A | P | G |
|  | G | 1.51 | 1.01 | **2.41** | **1.67** | 0.69 | 0.72 | 0.85 | **0.34** | 0.52 | 1.00 | 2.17 | 0.38 | 0.35 | 0.41 | 0.64 | 1.04 | 0.75 | 0.80 | 1.06 | 1.00 |
|  | P | 1.52 | 1.12 | **2.82** | **2.61** | 1.07 | 0.88 | 0.62 | **0.34** | **3.80** | 1.00 | 1.60 | 0.75 | 1.33 | 0.90 | 0.38 | **0.29** | 0.67 | **0.55** | 1.00 |  |
|  | A | **1.55** | 1.21 | **2.93** | **2.28** | 0.88 | 0.89 | 1.15 | **0.53** | 1.07 | 0.50 | 1.00 | **0.41** | **0.31** | 0.73 | **0.57** | **0.61** | **0.83** | 1.00 |  |  |
|  | V | **1.53** | 1.17 | **2.05** | **2.10** | 1.00 | 0.80 | 1.23 | 1.26 | 1.42 | 0.90 | 1.38 | 0.85 | 0.70 | **0.59** | **0.62** | **0.57** | 1.00 |  |  |  |
|  | L | **2.04** | 1.73 | **5.60** | **3.12** | 0.67 | 0.89 | **2.13** | 1.60 | 1.67 | 0.80 | 1.49 | 1.17 | 0.61 | **0.90** | 1.25 | 1.00 |  |  |  |  |
|  | I | **2.53** | 1.58 | **4.20** | **2.75** | 0.70 | 1.62 | 1.62 | 1.43 | 1.09 | 0.67 | **4.00** | 0.88 | 1.40 | 0.93 | 1.00 |  |  |  |  |  |
|  | M | 1.14 | 1.31 | 2.50 | **3.62** | 1.60 | 1.44 | 2.50 | 1.33 | 1.25 | 3.00 | 2.71 | 1.21 | 0.50 | 1.00 |  |  |  |  |  |  |
|  | C | 2.36 | 0.65 | 3.00 | - | 4.00 | 1.17 | 3.00 | 1.50 | 0.57 | - | 2.00 | 2.20 | 1.00 |  |  |  |  |  |  |  |
|  | F | 1.42 | 1.27 | **1.86** | 1.15 | 1.75 | 1.29 | 1.31 | 1.00 | 1.05 | 1.17 | 1.24 | 1.00 |  |  |  |  |  |  |  |  |
|  | Y | 1.15 | 0.69 | **3.38** | 1.33 | 1.08 | 0.93 | 1.05 | 0.93 | 1.36 | 0.63 | 1.00 |  |  |  |  |  |  |  |  |  |
|  | W | 3.00 | 1.00 | 3.50 | 2.20 | 0.67 | 1.00 | 1.50 | 1.67 | 0.83 | 1.00 |  |  |  |  |  |  |  |  |  |  |
|  | H | 1.04 | 1.94 | **4.44** | **2.11** | 1.10 | 0.73 | 1.24 | 0.86 | 1.00 |  |  |  |  |  |  |  |  |  |  |  |
|  | K | **3.29** | **4.92** | **8.56** | **5.19** | 2.32 | 1.35 | 1.33 | 1.00 |  |  |  |  |  |  |  |  |  |  |  |  |
|  | R | **1.67** | 1.03 | **2.71** | **1.95** | 1.13 | 1.01 | 1.00 |  |  |  |  |  |  |  |  |  |  |  |  |  |
|  | Q | 1.94 | 1.59 | **5.11** | **2.44** | 1.19 | 1.00 |  |  |  |  |  |  |  |  |  |  |  |  |  |  |
|  | N | 1.26 | 0.97 | **2.35** | **1.98** | 1.00 |  |  |  |  |  |  |  |  |  |  |  |  |  |  |  |
|  | E | 0.64 | 0.65 | **1.25** | 1.00 |  |  |  |  |  |  |  |  |  |  |  |  |  |  |  |  |
|  | D | 0.55 | 0.53 | 1.00 |  |  |  |  |  |  |  |  |  |  |  |  |  |  |  |  |  |
|  | S | **1.46** | 1.00 |  |  |  |  |  |  |  |  |  |  |  |  |  |  |  |  |  |  |
|  | T | 1.00 |  |  |  |  |  |  |  |  |  |  |  |  |  |  |  |  |  |  |  |

Each element R_ij_ in the matrix represents the ratio of number of replacements of the residue i by the residue j in the forward direction (non-halophiles→halophiles) to that in the reverse direction. This means that if R_ij_ >1, the number of replacement (i)_non-halophiles_→(j)_Halophiles_  is higher than the number of replacement (j) _non-halophiles_ →(i) _Halophiles_ and if R_ij_ <1, the reverse is true. Bold ratios signifies the directional bias at p<10^-2^ respectively.
