# Supplementary material for: Molecular signature of hypersaline adaptation: insights from genome and proteome composition of halophilic prokaryotes
Source: Genome Biol. 2008 Apr 9;9(4):R70. doi: 10.1186/gb-2008-9-4-r70 (PMC2643941; doi:10.1186/gb-2008-9-4-r70)
Supplement: Additional data file 4 — Trends in amino acid replacements in non-halophilic M. thermophila and halophilic H. marismortui chromosome I orthologs. [file gb-2008-9-4-r70-S4.doc]

**Additional Data File 4:** Amino acid replacement matrix for *H. marismortui proteins and their M. thermophila* orthologs (Set III)

|  | HMAR1 (halophile) | | | | | | | | | | | | | | | | | | | | |
| --- | --- | --- | --- | --- | --- | --- | --- | --- | --- | --- | --- | --- | --- | --- | --- | --- | --- | --- | --- | --- | --- |
| MTHP (non-halophile) |  | T | S | D | E | N | Q | R | K | H | W | Y | F | C | M | I | L | V | A | P | G |
|  | G | 1.37 | 0.72 | **2.35** | 1.12 | 0.87 | 1.13 | **0.51** | **0.25** | 1.09 | 1.00 | 0.69 | 0.63 | 0.43 | **0.36** | 0.59 | **0.55** | 0.77 | 1.07 | 0.69 | 1.00 |
|  | P | 1.59 | 0.84 | **2.35** | **1.45** | 0.93 | 1.22 | **0.53** | **0.46** | 1.00 | 1.00 | 0.89 | 0.71 | 0.42 | 0.45 | **0.49** | **0.56** | 0.84 | **1.56** | 1 .00 |  |
|  | A | 1.22 | **0.62** | **1.33** | 0.83 | 0.71 | 1.16 | **0.47** | **0.35** | 0.83 | 0.87 | **0.53** | 0.61 | **0.44** | **0.41** | **0.36** | **0.47** | **0.77** | 1.00 |  |  |
|  | V | **1.88** | 0.89 | **2.36** | **1.83** | 0.98 | 2.20 | 1.03 | 0.68 | 1.67 | 1.18 | 1.10 | 0.77 | 0.75 | **0.61** | **0.45** | **0.63** | 1.00 |  |  |  |
|  | L | **2.54** | 1.33 | **4.49** | **2.82** | 2.00 | **2.56** | **1.98** | 1.17 | 2.59 | 1.69 | 1.45 | **1.33** | 1.01 | **0.72** | **0.59** | 1.00 |  |  |  |  |
|  | I | **3.84** | **1.94** | **4.80** | **3.38** | 2.08 | **5.10** | **2.54** | 1.59 | **2.72** | **3.88** | **2.76** | **2.02** | 2.41 | 1.34 | 1.00 |  |  |  |  |  |
|  | M | **2.85** | 1.41 | **7.28** | **3.81** | 2.59 | **4.91** | **3.68** | 2.18 | **4.44** | 2.62 | 1.76 | 1.48 | 0.69 | 1.00 |  |  |  |  |  |  |
|  | C | **3.28** | 1.50 | **5.31** | **4.86** | 4.00 | 10.00 | 2.35 | 2.33 | 2.17 | 1.50 | 3.07 | 1.81 | 1.00 |  |  |  |  |  |  |  |
|  | F | 1.64 | 1.46 | **3.27** | 1.30 | 1.56 | 2.38 | 1.04 | 0.64 | 1.74 | 1.15 | 1.10 | 1.00 |  |  |  |  |  |  |  |  |
|  | Y | **2.18** | 1.07 | **3.36** | 1.67 | 2.17 | 2.08 | 1.07 | 0.63 | 1.86 | 1.47 | 1.00 |  |  |  |  |  |  |  |  |  |
|  | W | 1.20 | 1.03 | 3.69 | 1.83 | 1.27 | 1.85 | 1.16 | 0.94 | 2.39 | 1.00 |  |  |  |  |  |  |  |  |  |  |
|  | H | 1.42 | 0.64 | **1.67** | 1.48 | 0.96 | 1.10 | **0.64** | **0.37** | 1.00 |  |  |  |  |  |  |  |  |  |  |  |
|  | K | **3.79** | **2.05** | **7.30** | **4.11** | **2.50** | **4.42** | 1.09 | 1.00 |  |  |  |  |  |  |  |  |  |  |  |  |
|  | R | **3.10** | **1.34** | **4.34** | **2.62** | **1.78** | 2.45 | 1.00 |  |  |  |  |  |  |  |  |  |  |  |  |  |
|  | Q | 1.12 | **0.52** | **1.57** | 0.95 | 0.62 | 1.00 |  |  |  |  |  |  |  |  |  |  |  |  |  |  |
|  | N | **1.69** | 0.79 | **2.23** | **1.58** | 1.00 |  |  |  |  |  |  |  |  |  |  |  |  |  |  |  |
|  | E | 1.26 | **0.50** | **1.52** | 1.00 |  |  |  |  |  |  |  |  |  |  |  |  |  |  |  |  |
|  | D | 0.87 | **0.35** | 1.00 |  |  |  |  |  |  |  |  |  |  |  |  |  |  |  |  |  |
|  | S | **1.53** | 1.00 |  |  |  |  |  |  |  |  |  |  |  |  |  |  |  |  |  |  |
|  | T | 1.00 |  |  |  |  |  |  |  |  |  |  |  |  |  |  |  |  |  |  |  |

Each element R_ij_ in the matrix represents the ratio of number of replacements of the residue i by the residue j in the forward direction (non-halophiles→halophiles) to that in the reverse direction. This means that if R_ij_ >1, the number of replacement (i)_non-halophiles_→(j)_Halophiles_  is higher than the number of replacement (j) _non-halophiles_ →(i) _Halophiles_ and if R_ij_ <1, the reverse is true. Bold ratios signifies the directional bias at p<10^-6^ respectively.
