# Supplementary material for: Molecular signature of hypersaline adaptation: insights from genome and proteome composition of halophilic prokaryotes
Source: Genome Biol. 2008 Apr 9;9(4):R70. doi: 10.1186/gb-2008-9-4-r70 (PMC2643941; doi:10.1186/gb-2008-9-4-r70)
Supplement: Additional data file 5 — Trends in amino acid replacements in non-halophilic M. archaeon and halophilic N. pharaonis orthologs. [file gb-2008-9-4-r70-S5.doc]

**Additional Data File 5:** Amino acid replacement matrix for *N. pharaonis proteins and their methanogenic archaeon* orthologs (Set IV)

|  | NPHA (halophile) | | | | | | | | | | | | | | | | | | | | |
| --- | --- | --- | --- | --- | --- | --- | --- | --- | --- | --- | --- | --- | --- | --- | --- | --- | --- | --- | --- | --- | --- |
| UMET (non-halophile) |  | T | S | D | E | N | Q | R | K | H | W | Y | F | C | M | I | L | V | A | P | G |
|  | G | 0.97 | 0.72 | **1.85** | 1.30 | **0.55** | 0.66 | 0.76 | **0.15** | 0.69 | 0.68 | 0.41 | 0.47 | 0.42 | **0.32** | **0.35** | **0.52** | 0.76 | 1.09 | 0.88 | 1.00 |
|  | P | 1.02 | 1.01 | **2.01** | **1.63** | 0.58 | 0.74 | 0.97 | **0.20** | 1.02 | 0.94 | 0.39 | 0.54 | 0.35 | **0.29** | **0.41** | **0.55** | 0.66 | 1.10 | 1.00 |  |
|  | A | 0.81 | **0.61** | **1.33** | 0.90 | 0.61 | 0.67 | 0.77 | **0.12** | 0.76 | 0.90 | **0.38** | 0.60 | **0.35** | **0.32** | **0.36** | **0.56** | **0.71** | 1.00 |  |  |
|  | V | **1.31** | 0.96 | **1.97** | **2.01** | 0.8 | 1.15 | 1.59 | **0.41** | 1.09 | 1.24 | 0.83 | **0.64** | 0.77 | **0.47** | **0.39** | **0.76** | 1.00 |  |  |  |
|  | L | **1.54** | 1.29 | **3.23** | **3.08** | 1.04 | 1.61 | **2.79** | **0.55** | 2.13 | 1.50 | 0.89 | 0.92 | 1.24 | **0.52** | **0.51** | 1.00 |  |  |  |  |
|  | I | **3.88** | 1.73 | **4.05** | **4.75** | 1.46 | **2.88** | **3.81** | 0.98 | **4.48** | 2.88 | 1.55 | 1.76 | 1.27 | 1.10 | 1.00 |  |  |  |  |  |
|  | M | **2.41** | 1.72 | **6.50** | **3.48** | 1.61 | **3.26** | **5.59** | 0.94 | **3.82** | 1.92 | 1.54 | 1.51 | 1.55 | 1.00 |  |  |  |  |  |  |
|  | C | 2.07 | 1.37 | 3.31 | 3.25 | 4.00 | 3.20 | **5.00** | 1.23 | 2.25 | 2.33 | 1.56 | 1.52 | 1.00 |  |  |  |  |  |  |  |
|  | F | 1.82 | 1.18 | **2.53** | 2.04 | 0.8 | 1.38 | **2.79** | 0.62 | 2.23 | 1.77 | 0.96 | 1.00 |  |  |  |  |  |  |  |  |
|  | Y | **2.04** | 1.21 | **3.02** | **2.66** | 1.16 | 1.17 | 2.53 | 0.49 | **3.25** | 1.79 | 1.00 |  |  |  |  |  |  |  |  |  |
|  | W | 1.41 | 0.52 | 2.00 | 1.36 | 0.80 | 1.00 | 1.20 | 0.30 | 1.81 | 1.00 |  |  |  |  |  |  |  |  |  |  |
|  | H | 1.02 | 1.16 | 1.62 | **1.82** | 0.76 | 1.09 | 1.18 | **0.35** | 1.00 |  |  |  |  |  |  |  |  |  |  |  |
|  | K | **5.44** | **4.42** | **12.93** | **8.72** | **2.77** | **3.95** | **3.01** | 1.00 |  |  |  |  |  |  |  |  |  |  |  |  |
|  | R | 1.11 | 1.01 | **2.00** | **1.57** | 0.60 | 0.94 | 1.00 |  |  |  |  |  |  |  |  |  |  |  |  |  |
|  | Q | 1.20 | 1.24 | **2.56** | **1.88** | 0.67 | 1.00 |  |  |  |  |  |  |  |  |  |  |  |  |  |  |
|  | N | **1.89** | 1.41 | **3.20** | **2.65** | 1.00 |  |  |  |  |  |  |  |  |  |  |  |  |  |  |  |
|  | E | **0.72** | **0.68** | **1.23** | 1.00 |  |  |  |  |  |  |  |  |  |  |  |  |  |  |  |  |
|  | D | **0.57** | **0.50** | 1.00 |  |  |  |  |  |  |  |  |  |  |  |  |  |  |  |  |  |
|  | S | 1.19 | 1.00 |  |  |  |  |  |  |  |  |  |  |  |  |  |  |  |  |  |  |
|  | T | 1.00 |  |  |  |  |  |  |  |  |  |  |  |  |  |  |  |  |  |  |  |

Each element R_ij_ in the matrix represents the ratio of number of replacements of the residue i by the residue j in the forward direction (non-halophiles→halophiles) to that in the reverse direction. This means that if R_ij_ >1, the number of replacement (i)_non-halophiles_→(j)_Halophiles_  is higher than the number of replacement (j) _non-halophiles_ →(i) _Halophiles_ and if R_ij_ <1, the reverse is true. Bold ratios signifies the directional bias at p<10^-6^ respectively.
