# Supplementary material for: Molecular signature of hypersaline adaptation: insights from genome and proteome composition of halophilic prokaryotes
Source: Genome Biol. 2008 Apr 9;9(4):R70. doi: 10.1186/gb-2008-9-4-r70 (PMC2643941; doi:10.1186/gb-2008-9-4-r70)
Supplement: Additional data file 6 — Number of amino acid replacements from non-halophilic P. luteolum to halophilic S. ruber orthologs. [file gb-2008-9-4-r70-S6.doc]

**Additional Data File 6:** Number of replacements between each residue pairs of *S. ruber* proteins and their *P. luteolum* orthologs (Set I)

|  | SRUB (halophile) | | | | | | | | | | | | | | | | | | | | |
| --- | --- | --- | --- | --- | --- | --- | --- | --- | --- | --- | --- | --- | --- | --- | --- | --- | --- | --- | --- | --- | --- |
| PLUT (non-halophile) |  | G | P | A | V | L | I | M | C | F | Y | W | H | K | R | Q | N | E | D | S | T |
|  | G | 6017 | 86 | 435 | 36 | 37 | 10 | 15 | 18 | 19 | 16 | 4 | 53 | 61 | 138 | 83 | 121 | 321 | 485 | 227 | 113 |
|  | P | 91 | 2941 | 235 | 55 | 54 | 7 | 14 | 3 | 18 | 12 | 3 | 18 | 32 | 78 | 45 | 24 | 196 | 233 | 137 | 94 |
|  | A | 499 | 301 | 4686 | 460 | 220 | 102 | 76 | 103 | 38 | 44 | 17 | 83 | 116 | 279 | 179 | 101 | 555 | 415 | 534 | 394 |
|  | V | 50 | 113 | 539 | 3916 | 635 | 895 | 146 | 57 | 74 | 44 | 14 | 30 | 70 | 132 | 74 | 35 | 206 | 72 | 80 | 328 |
|  | L | 67 | 106 | 308 | 833 | 5279 | 647 | 390 | 39 | 304 | 98 | 49 | 92 | 80 | 252 | 142 | 29 | 201 | 107 | 68 | 196 |
|  | I | 27 | 59 | 202 | 1632 | 1195 | 2691 | 233 | 29 | 126 | 50 | 9 | 45 | 44 | 105 | 52 | 24 | 94 | 34 | 34 | 209 |
|  | M | 19 | 48 | 110 | 205 | 522 | 178 | 1056 | 13 | 80 | 31 | 20 | 27 | 53 | 112 | 60 | 22 | 95 | 39 | 50 | 126 |
|  | C | 35 | 12 | 135 | 74 | 35 | 15 | 7 | 385 | 15 | 22 | 4 | 9 | 5 | 24 | 9 | 12 | 18 | 27 | 47 | 55 |
|  | F | 19 | 38 | 87 | 152 | 379 | 106 | 77 | 14 | 1964 | 417 | 58 | 85 | 17 | 56 | 31 | 10 | 39 | 34 | 38 | 55 |
|  | Y | 17 | 19 | 59 | 67 | 105 | 34 | 27 | 5 | 282 | 1611 | 50 | 190 | 13 | 52 | 38 | 23 | 42 | 29 | 29 | 53 |
|  | W | 5 | 9 | 17 | 7 | 12 | 4 | 7 | 3 | 38 | 39 | 471 | 15 | 8 | 15 | 5 | 1 | 14 | 1 | 10 | 4 |
|  | H | 57 | 38 | 81 | 34 | 55 | 12 | 15 | 4 | 26 | 86 | 11 | 1127 | 44 | 135 | 77 | 65 | 119 | 130 | 53 | 72 |
|  | K | 167 | 140 | 287 | 78 | 74 | 35 | 32 | 4 | 16 | 26 | 9 | 92 | 1725 | 856 | 330 | 124 | 704 | 438 | 196 | 230 |
|  | R | 147 | 115 | 262 | 92 | 95 | 17 | 38 | 11 | 22 | 53 | 16 | 140 | 416 | 3497 | 372 | 125 | 497 | 308 | 156 | 217 |
|  | Q | 88 | 71 | 172 | 51 | 63 | 20 | 32 | 4 | 10 | 14 | 8 | 86 | 116 | 265 | 1174 | 51 | 444 | 256 | 104 | 113 |
|  | N | 133 | 34 | 113 | 24 | 42 | 18 | 16 | 10 | 24 | 19 | 6 | 129 | 68 | 139 | 93 | 1359 | 212 | 431 | 175 | 132 |
|  | E | 204 | 202 | 510 | 106 | 69 | 31 | 29 | 4 | 23 | 31 | 8 | 117 | 172 | 357 | 409 | 115 | 3960 | 1214 | 221 | 262 |
|  | D | 200 | 103 | 235 | 39 | 24 | 4 | 15 | 3 | 14 | 18 | 3 | 79 | 69 | 120 | 133 | 176 | 874 | 3403 | 171 | 170 |
|  | S | 287 | 179 | 818 | 123 | 84 | 24 | 38 | 32 | 31 | 44 | 8 | 110 | 115 | 240 | 144 | 183 | 403 | 438 | 2094 | 616 |
|  | T | 102 | 132 | 370 | 283 | 118 | 82 | 60 | 28 | 35 | 27 | 10 | 64 | 87 | 206 | 110 | 85 | 324 | 209 | 405 | 2514 |

The value in each cell (i, j) indicates the number of times the amino acid residue for the i-th row in non-halophilic orthologous proteins replaced by the amino acid residue for the j-th column in the halophilic protein.
