# Supplementary material for: Molecular signature of hypersaline adaptation: insights from genome and proteome composition of halophilic prokaryotes
Source: Genome Biol. 2008 Apr 9;9(4):R70. doi: 10.1186/gb-2008-9-4-r70 (PMC2643941; doi:10.1186/gb-2008-9-4-r70)
Supplement: Additional data file 7 — Number of amino acid replacements from non-halophilic P. putida to halophilic H. marismortui chromosome I orthologs. [file gb-2008-9-4-r70-S7.doc]

**Additional Data File 7:** Number of replacements between each residue pairs of *H. marismortui* (ch-I) and *their P. putida orthologs* (Set II)

|  | HMAR1 (halophile) | | | | | | | | | | | | | | | | | | | | |
| --- | --- | --- | --- | --- | --- | --- | --- | --- | --- | --- | --- | --- | --- | --- | --- | --- | --- | --- | --- | --- | --- |
| PPUT (non-halophile) |  | G | P | A | V | L | I | M | C | F | Y | W | H | K | R | Q | N | E | D | S | T |
|  | G | 2301 | 38 | 205 | 30 | 28 | 9 | 7 | 7 | 3 | 13 | 4 | 17 | 22 | 51 | 43 | 42 | 135 | 210 | 101 | 56 |
|  | P | 36 | 1030 | 71 | 36 | 14 | 8 | 9 | 4 | 6 | 8 | 3 | 19 | 11 | 24 | 21 | 15 | 99 | 107 | 55 | 50 |
|  | A | 257 | 130 | 1754 | 201 | 96 | 52 | 37 | 25 | 16 | 34 | 6 | 46 | 54 | 129 | 86 | 45 | 351 | 264 | 246 | 198 |
|  | V | 40 | 54 | 241 | 1339 | 264 | 321 | 51 | 28 | 60 | 36 | 9 | 27 | 34 | 58 | 33 | 22 | 101 | 45 | 54 | 170 |
|  | L | 27 | 48 | 157 | 462 | 1719 | 349 | 156 | 11 | 148 | 64 | 12 | 40 | 40 | 100 | 49 | 12 | 100 | 56 | 38 | 106 |
|  | I | 14 | 21 | 92 | 521 | 280 | 736 | 65 | 14 | 50 | 40 | 6 | 12 | 20 | 34 | 26 | 14 | 44 | 21 | 19 | 96 |
|  | M | 17 | 10 | 51 | 86 | 173 | 70 | 342 | 3 | 35 | 19 | 6 | 10 | 20 | 20 | 26 | 8 | 29 | 20 | 17 | 33 |
|  | C | 20 | 3 | 81 | 40 | 18 | 10 | 6 | 146 | 11 | 6 | 0 | 4 | 3 | 9 | 7 | 8 | 9 | 12 | 15 | 33 |
|  | F | 8 | 8 | 39 | 71 | 126 | 57 | 29 | 5 | 555 | 134 | 27 | 20 | 6 | 21 | 9 | 7 | 15 | 13 | 19 | 17 |
|  | Y | 6 | 5 | 34 | 26 | 43 | 10 | 7 | 3 | 108 | 487 | 17 | 45 | 13 | 20 | 14 | 13 | 24 | 27 | 11 | 15 |
|  | W | 4 | 3 | 12 | 10 | 15 | 9 | 2 | 1 | 23 | 27 | 168 | 5 | 5 | 9 | 5 | 2 | 11 | 7 | 7 | 12 |
|  | H | 33 | 5 | 43 | 19 | 24 | 11 | 8 | 7 | 19 | 33 | 6 | 369 | 19 | 41 | 33 | 34 | 74 | 80 | 35 | 24 |
|  | K | 65 | 32 | 102 | 27 | 25 | 14 | 15 | 2 | 6 | 14 | 3 | 22 | 409 | 156 | 73 | 44 | 306 | 214 | 59 | 102 |
|  | R | 60 | 39 | 112 | 47 | 47 | 21 | 8 | 3 | 16 | 19 | 6 | 33 | 117 | 983 | 112 | 51 | 207 | 160 | 70 | 102 |
|  | Q | 60 | 24 | 97 | 41 | 55 | 16 | 18 | 6 | 7 | 15 | 5 | 45 | 54 | 111 | 443 | 38 | 266 | 189 | 59 | 62 |
|  | N | 61 | 14 | 51 | 22 | 18 | 20 | 5 | 2 | 4 | 12 | 3 | 31 | 19 | 45 | 32 | 461 | 95 | 169 | 62 | 67 |
|  | E | 81 | 38 | 154 | 48 | 32 | 16 | 8 | 0 | 13 | 18 | 5 | 35 | 59 | 106 | 109 | 48 | 1164 | 429 | 81 | 78 |
|  | D | 87 | 38 | 90 | 22 | 10 | 5 | 8 | 4 | 7 | 8 | 2 | 18 | 25 | 59 | 37 | 72 | 343 | 1207 | 80 | 60 |
|  | S | 100 | 49 | 203 | 46 | 22 | 12 | 13 | 23 | 15 | 16 | 7 | 18 | 12 | 68 | 37 | 64 | 124 | 152 | 643 | 249 |
|  | T | 37 | 33 | 128 | 111 | 52 | 38 | 29 | 14 | 12 | 13 | 4 | 23 | 31 | 61 | 32 | 53 | 122 | 109 | 170 | 844 |

The value in each cell (i, j) indicates the number of times the amino acid residue for the i-th row in non-halophilic orthologous proteins replaced by the amino acid residue for the j-th column in the halophilic protein.
