# Supplementary material for: Molecular signature of hypersaline adaptation: insights from genome and proteome composition of halophilic prokaryotes
Source: Genome Biol. 2008 Apr 9;9(4):R70. doi: 10.1186/gb-2008-9-4-r70 (PMC2643941; doi:10.1186/gb-2008-9-4-r70)
Supplement: Additional data file 8 — Number of amino acid replacements from non-halophilic M. thermophila to halophilic H. marismortui chromosome I orthologs. [file gb-2008-9-4-r70-S8.doc]

**Additional Data File 8:** Number of replacements between each residue pairs of *H. marismortui* (ch-I) proteins and their *M. thermophila* orthologs (Set III)

|  | HMAR1 (halophile) | | | | | | | | | | | | | | | | | | | | |
| --- | --- | --- | --- | --- | --- | --- | --- | --- | --- | --- | --- | --- | --- | --- | --- | --- | --- | --- | --- | --- | --- |
| MTHP (non-halophile) |  | G | P | A | V | L | I | M | C | F | Y | W | H | K | R | Q | N | E | D | S | T |
|  | G | 9193 | 176 | 1024 | 151 | 119 | 64 | 39 | 40 | 45 | 60 | 24 | 108 | 82 | 281 | 145 | 222 | 617 | 1183 | 497 | 280 |
|  | P | 255 | 4666 | 547 | 187 | 129 | 80 | 38 | 15 | 40 | 56 | 19 | 65 | 74 | 174 | 115 | 69 | 470 | 522 | 264 | 271 |
|  | A | 956 | 351 | 7358 | 1053 | 448 | 274 | 148 | 142 | 132 | 114 | 45 | 148 | 176 | 458 | 277 | 186 | 896 | 728 | 967 | 790 |
|  | V | 197 | 223 | 1376 | 6384 | 1237 | 1513 | 251 | 126 | 271 | 194 | 45 | 120 | 130 | 348 | 178 | 90 | 501 | 285 | 279 | 892 |
|  | L | 215 | 232 | 946 | 1978 | 7513 | 1230 | 620 | 98 | 717 | 324 | 83 | 192 | 185 | 558 | 264 | 124 | 494 | 337 | 232 | 561 |
|  | I | 108 | 164 | 756 | 3368 | 2087 | 3681 | 407 | 77 | 379 | 207 | 62 | 106 | 126 | 323 | 148 | 75 | 318 | 192 | 186 | 572 |
|  | M | 109 | 85 | 363 | 413 | 863 | 303 | 1380 | 18 | 167 | 95 | 34 | 80 | 109 | 239 | 157 | 57 | 259 | 182 | 131 | 254 |
|  | C | 93 | 36 | 325 | 169 | 97 | 32 | 26 | 774 | 38 | 43 | 9 | 26 | 21 | 47 | 30 | 32 | 68 | 69 | 120 | 131 |
|  | F | 71 | 56 | 218 | 352 | 538 | 188 | 113 | 21 | 2304 | 524 | 105 | 122 | 27 | 100 | 62 | 42 | 83 | 98 | 83 | 105 |
|  | Y | 87 | 63 | 217 | 176 | 223 | 75 | 54 | 14 | 478 | 2174 | 113 | 260 | 46 | 159 | 77 | 63 | 137 | 158 | 103 | 133 |
|  | W | 24 | 19 | 52 | 38 | 49 | 16 | 13 | 6 | 91 | 77 | 661 | 43 | 15 | 79 | 24 | 14 | 44 | 48 | 33 | 24 |
|  | H | 99 | 65 | 179 | 72 | 74 | 39 | 18 | 12 | 70 | 140 | 18 | 1629 | 55 | 188 | 119 | 129 | 224 | 237 | 112 | 119 |
|  | K | 331 | 162 | 501 | 191 | 158 | 79 | 50 | 9 | 42 | 73 | 16 | 150 | 1829 | 916 | 407 | 210 | 1162 | 883 | 357 | 425 |
|  | R | 550 | 327 | 978 | 337 | 282 | 127 | 65 | 20 | 96 | 149 | 68 | 295 | 842 | 5444 | 697 | 312 | 1836 | 1306 | 648 | 740 |
|  | Q | 128 | 94 | 239 | 81 | 103 | 29 | 32 | 3 | 26 | 37 | 13 | 108 | 92 | 284 | 1346 | 78 | 584 | 359 | 151 | 183 |
|  | N | 255 | 74 | 261 | 92 | 62 | 36 | 22 | 8 | 27 | 29 | 11 | 134 | 84 | 175 | 125 | 1672 | 387 | 753 | 261 | 236 |
|  | E | 550 | 325 | 1081 | 274 | 175 | 94 | 68 | 14 | 64 | 82 | 24 | 151 | 283 | 701 | 613 | 245 | 6292 | 2576 | 527 | 633 |
|  | D | 504 | 222 | 546 | 121 | 75 | 40 | 25 | 13 | 30 | 47 | 13 | 142 | 121 | 301 | 228 | 337 | 1693 | 5766 | 462 | 370 |
|  | S | 689 | 316 | 1556 | 312 | 175 | 96 | 93 | 80 | 57 | 96 | 32 | 174 | 174 | 482 | 292 | 330 | 1054 | 1312 | 2959 | 1048 |
|  | T | 204 | 170 | 645 | 475 | 221 | 149 | 89 | 40 | 64 | 61 | 20 | 84 | 112 | 239 | 164 | 140 | 502 | 423 | 683 | 3368 |

The value in each cell (i, j) indicates the number of times the amino acid residue for the i-th row in non-halophilic orthologous proteins replaced by the amino acid residue for the j-th column in the halophilic protein.
