# Supplementary material for: Molecular signature of hypersaline adaptation: insights from genome and proteome composition of halophilic prokaryotes
Source: Genome Biol. 2008 Apr 9;9(4):R70. doi: 10.1186/gb-2008-9-4-r70 (PMC2643941; doi:10.1186/gb-2008-9-4-r70)
Supplement: Additional data file 9 — Number of amino acid replacements from non-halophilic M. archaeon to halophilic N. pharaonis orthologs. [file gb-2008-9-4-r70-S9.doc]

**Additional Data File 9:** Number of replacements between each residue pairs of *N. pharaonis* proteins and their *methanogenic archaeon* orthologs (Set IV)

|  | NPHA (halophile) | | | | | | | | | | | | | | | | | | | | |
| --- | --- | --- | --- | --- | --- | --- | --- | --- | --- | --- | --- | --- | --- | --- | --- | --- | --- | --- | --- | --- | --- |
| UMET (non-halophile) |  | G | P | A | V | L | I | M | C | F | Y | W | H | K | R | Q | N | E | D | S | T |
|  | G | 9388 | 212 | 1075 | 162 | 97 | 44 | 37 | 36 | 38 | 37 | 19 | 83 | 79 | 285 | 122 | 215 | 601 | 1037 | 445 | 282 |
|  | P | 240 | 4628 | 506 | 174 | 127 | 65 | 22 | 13 | 45 | 29 | 17 | 52 | 61 | 212 | 95 | 66 | 497 | 505 | 289 | 246 |
|  | A | 988 | 459 | 7638 | 1040 | 505 | 273 | 122 | 144 | 132 | 112 | 38 | 136 | 120 | 530 | 245 | 190 | 1066 | 863 | 920 | 739 |
|  | V | 214 | 264 | 1464 | 6468 | 1329 | 1389 | 216 | 136 | 241 | 176 | 46 | 102 | 115 | 378 | 123 | 75 | 519 | 262 | 260 | 817 |
|  | L | 186 | 230 | 905 | 1753 | 7359 | 1096 | 517 | 88 | 597 | 281 | 90 | 168 | 136 | 552 | 205 | 88 | 453 | 252 | 198 | 481 |
|  | I | 127 | 160 | 760 | 3584 | 2135 | 3656 | 340 | 65 | 345 | 170 | 46 | 103 | 88 | 278 | 124 | 67 | 318 | 174 | 152 | 606 |
|  | M | 114 | 75 | 382 | 464 | 1000 | 308 | 1386 | 34 | 156 | 83 | 23 | 65 | 72 | 246 | 153 | 45 | 212 | 130 | 119 | 248 |
|  | C | 86 | 37 | 410 | 176 | 71 | 51 | 22 | 744 | 38 | 42 | 7 | 18 | 16 | 60 | 16 | 32 | 52 | 53 | 92 | 126 |
|  | F | 80 | 84 | 219 | 375 | 650 | 196 | 103 | 25 | 2414 | 554 | 113 | 134 | 31 | 148 | 55 | 28 | 112 | 101 | 72 | 153 |
|  | Y | 90 | 74 | 297 | 213 | 314 | 110 | 54 | 27 | 578 | 2321 | 122 | 358 | 49 | 258 | 68 | 71 | 197 | 145 | 94 | 167 |
|  | W | 28 | 18 | 42 | 37 | 60 | 16 | 12 | 3 | 64 | 68 | 644 | 29 | 8 | 55 | 16 | 12 | 45 | 32 | 11 | 31 |
|  | H | 120 | 51 | 179 | 94 | 79 | 23 | 17 | 8 | 60 | 110 | 16 | 1597 | 53 | 219 | 121 | 105 | 255 | 228 | 125 | 115 |
|  | K | 519 | 300 | 993 | 283 | 247 | 90 | 77 | 13 | 50 | 101 | 27 | 151 | 2037 | 1623 | 510 | 285 | 2023 | 1461 | 570 | 685 |
|  | R | 374 | 219 | 689 | 237 | 198 | 73 | 44 | 12 | 53 | 102 | 46 | 186 | 540 | 4905 | 403 | 179 | 1136 | 754 | 431 | 468 |
|  | Q | 185 | 128 | 367 | 107 | 127 | 43 | 47 | 5 | 40 | 58 | 16 | 111 | 129 | 428 | 1377 | 90 | 807 | 541 | 218 | 191 |
|  | N | 389 | 113 | 311 | 94 | 85 | 46 | 28 | 8 | 35 | 61 | 15 | 138 | 103 | 300 | 134 | 1702 | 565 | 1024 | 355 | 284 |
|  | E | 461 | 304 | 1179 | 258 | 147 | 67 | 61 | 16 | 55 | 74 | 33 | 140 | 232 | 724 | 429 | 213 | 5990 | 2181 | 550 | 541 |
|  | D | 561 | 251 | 647 | 133 | 78 | 43 | 20 | 16 | 40 | 48 | 16 | 141 | 113 | 377 | 211 | 320 | 1774 | 5752 | 462 | 375 |
|  | S | 617 | 286 | 1515 | 272 | 153 | 88 | 69 | 67 | 61 | 78 | 21 | 108 | 129 | 425 | 176 | 251 | 812 | 915 | 2861 | 942 |
|  | T | 290 | 240 | 916 | 622 | 313 | 156 | 103 | 61 | 84 | 82 | 22 | 113 | 126 | 422 | 159 | 150 | 754 | 663 | 793 | 3790 |

The value in each cell (i, j) indicates the number of times the amino acid residue for the i-th row in non-halophilic orthologous proteins replaced by the amino acid residue for the j-th column in the halophilic protein.
