# Supplementary material for: Molecular signature of hypersaline adaptation: insights from genome and proteome composition of halophilic prokaryotes
Source: Genome Biol. 2008 Apr 9;9(4):R70. doi: 10.1186/gb-2008-9-4-r70 (PMC2643941; doi:10.1186/gb-2008-9-4-r70)
Supplement: Additional data file 10 — Dinucleotide relative abundance for all the organisms under study. [file gb-2008-9-4-r70-S10.doc]

| Organisms | Dinucleotides | | | | | | | | | |
| --- | --- | --- | --- | --- | --- | --- | --- | --- | --- | --- |
|  | AA  TT | AC  GT | AG  CT | AT | CA  TG | CC  GG | CG | GA  TC | GC | TA |
|  |  |  |  |  |  |  |  |  |  |  |
| HALO | 0.92 | 1.26 | 0.79 | 0.97 | 0.92 | 0.77 | **1.36** | **1.33** | 0.94 | **0.54** |
| HMAR1 | 0.95 | 1.16 | 0.89 | 0.97 | 0.91 | 0.79 | **1.32** | **1.33** | 0.90 | **0.64** |
| HMAR2 | 0.96 | 1.11 | 0.92 | 1.01 | 1.00 | 0.82 | 1.24 | 1.26 | 0.90 | **0.68** |
| HSAL | 0.92 | 1.26 | 0.79 | 0.97 | 0.92 | 0.77 | **1.36** | **1.33** | 0.94 | **0.54** |
| HWAL | 0.94 | 0.98 | 0.87 | 1.19 | 1.19 | 0.82 | 1.11 | 1.23 | 0.94 | **0.68** |
| NPHA | 1.03 | 1.12 | 0.86 | 1.00 | 0.83 | 0.78 | **1.39** | **1.36** | 0.93 | **0.64** |
| SRUB | 1.08 | 1.02 | 0.93 | 1.02 | 0.93 | 0.93 | 1.23 | 1.26 | 0.92 | **0.54** |
|  |  |  |  |  |  |  |  |  |  |  |
| ABAC | 1.20 | 0.89 | 0.88 | 1.12 | 1.02 | 0.80 | 1.27 | 1.18 | 1.15 | **0.53** |
| APER | 0.88 | 0.83 | 1.31 | 0.95 | 0.90 | 1.14 | **0.70** | 1.03 | 0.96 | 1.21 |
| AZOA | 1.15 | 0.89 | 0.87 | 1.28 | 1.00 | 0.73 | **1.33** | 1.22 | 1.21 | **0.44** |
| BLON | 1.06 | 0.95 | 0.81 | 1.29 | 1.13 | 0.86 | 1.17 | 1.11 | 1.10 | **0.57** |
| CCRE | 1.09 | 0.86 | 0.96 | 1.29 | 1.01 | 0.85 | 1.16 | 1.21 | 1.11 | **0.45** |
| ECOL | 1.21 | 0.88 | 0.82 | 1.10 | 1.12 | 0.90 | 1.15 | 0.92 | 1.28 | 0.75 |
| GVIO | 1.28 | 0.85 | 0.93 | 1.07 | 1.05 | 0.93 | 1.08 | 1.04 | 1.13 | **0.57** |
| MTHA | 0.95 | 0.85 | 1.07 | 1.13 | 1.17 | 1.25 | **0.51** | 1.14 | 0.76 | 0.74 |
| MTHP | 0.82 | 0.74 | 1.14 | **1.32** | 1.11 | 0.92 | 0.86 | **1.33** | 1.02 | **0.67** |
| PCAL | 1.09 | 0.92 | 1.15 | 0.82 | 0.94 | 1.05 | 0.88 | 0.96 | 1.04 | 1.05 |
| PLUT | 1.14 | 0.81 | 0.96 | 1.17 | 1.07 | 0.96 | 1.01 | 1.17 | 1.05 | **0.54** |
| POLA | 1.25 | 0.85 | 0.89 | 1.17 | 1.26 | 0.88 | 1.02 | 0.92 | 1.25 | **0.45** |
| PPRO | 1.12 | 0.83 | 0.95 | 1.19 | 1.12 | 1.03 | 0.92 | 1.10 | 1.02 | **0.56** |
| PPUT | 1.13 | 0.91 | 0.95 | 1.09 | 1.26 | 0.89 | 0.97 | 0.92 | 1.21 | **0.56** |
| PTHE | **1.42** | 0.74 | 0.90 | 0.98 | 0.96 | 1.20 | 0.92 | 0.85 | 1.16 | 0.79 |
| RCAS | 1.07 | 0.88 | 0.81 | **1.39** | 1.13 | 0.77 | 1.26 | 1.11 | 1.23 | **0.55** |
| SBOY | 1.19 | 0.89 | 0.83 | 1.09 | 1.13 | 0.89 | 1.14 | 0.92 | 1.27 | 0.75 |
| SYNE | 1.11 | 0.82 | 1.02 | 1.13 | 1.27 | 0.93 | 0.88 | 1.12 | 1.11 | **0.30** |
| TACI | 0.97 | 0.75 | 0.97 | 1.26 | 1.06 | 1.05 | 0.91 | 1.17 | 1.04 | 0.82 |
| TKOD | 1.15 | 0.82 | 1.19 | 0.85 | 0.88 | 1.06 | 0.88 | 1.23 | 0.90 | 0.73 |
| TMAR | 1.19 | 0.87 | 1.11 | 0.83 | 0.97 | 0.99 | 0.92 | **1.40** | **0.69** | **0.50** |
| TPEN | 1.03 | 0.92 | 1.27 | 0.71 | 0.76 | 0.98 | 1.00 | 1.15 | 0.97 | 1.10 |
| UMET | 1.00 | 0.85 | 1.03 | 1.15 | 1.01 | 0.97 | 1.00 | 1.12 | 1.06 | 0.84 |
| YPES | 1.15 | 0.87 | 0.83 | 1.12 | 1.14 | 1.03 | 0.99 | 0.88 | 1.23 | 0.83 |

**Additional Data File 10:** Dinucleotide relative abundance values of six obligatory halophiles and twenty four non-halophiles

Significantly overrepresented dinucleotide relative abundance values are indicated in blue when abundance value is ≥ 1.23 (in bold face if ≥ 1.30), while significantly underrepresentation is shown in red when abundance value is ≤ 0.78 (in bold face if ≤ 0.70) (as described by Karlin et al., *Theoretical population biology*, 61, 367-390, 2002).
