# Supplementary material for: Molecular signature of hypersaline adaptation: insights from genome and proteome composition of halophilic prokaryotes
Source: Genome Biol. 2008 Apr 9;9(4):R70. doi: 10.1186/gb-2008-9-4-r70 (PMC2643941; doi:10.1186/gb-2008-9-4-r70)
Supplement: Additional data file 11 — Dinucleotide frequency at the first and second codon positions for all the organisms under study. [file gb-2008-9-4-r70-S11.doc]

**Additional Data File 11:** Dinucleotide frequency at 1^st^ and 2^nd^ codon position for all the organisms under study

|  | Organism | AA* | AC+ | AG* | AT* | CA | CC | CG | CT | GA+ | GC | GG | GT+ | TA | TC | TG* | TT* |
| --- | --- | --- | --- | --- | --- | --- | --- | --- | --- | --- | --- | --- | --- | --- | --- | --- | --- |
|  |  |  |  |  |  |  |  |  |  |  |  |  |  |  |  |  |  |
| Halophiles | HALO | 0.037 | 0.068 | 0.020 | 0.052 | 0.048 | 0.047 | 0.063 | 0.076 | 0.157 | 0.131 | 0.085 | 0.096 | 0.027 | 0.034 | 0.019 | 0.038 |
|  | HMAR1 | 0.044 | 0.068 | 0.023 | 0.061 | 0.050 | 0.046 | 0.057 | 0.082 | 0.166 | 0.107 | 0.084 | 0.088 | 0.028 | 0.038 | 0.020 | 0.038 |
|  | HMAR2 | 0.051 | 0.073 | 0.029 | 0.069 | 0.053 | 0.044 | 0.053 | 0.079 | 0.157 | 0.098 | 0.080 | 0.080 | 0.031 | 0.038 | 0.020 | 0.045 |
|  | HSAL | 0.032 | 0.060 | 0.021 | 0.043 | 0.055 | 0.055 | 0.091 | 0.071 | 0.146 | 0.126 | 0.092 | 0.099 | 0.021 | 0.035 | 0.020 | 0.033 |
|  | HWAL | 0.058 | 0.079 | 0.026 | 0.079 | 0.055 | 0.043 | 0.052 | 0.065 | 0.151 | 0.094 | 0.075 | 0.078 | 0.029 | 0.048 | 0.019 | 0.049 |
|  | NPHA | 0.040 | 0.064 | 0.020 | 0.058 | 0.045 | 0.046 | 0.060 | 0.081 | 0.176 | 0.115 | 0.084 | 0.089 | 0.028 | 0.036 | 0.019 | 0.038 |
|  | SRUB | 0.045 | 0.062 | 0.020 | 0.055 | 0.058 | 0.057 | 0.075 | 0.092 | 0.140 | 0.107 | 0.084 | 0.079 | 0.028 | 0.040 | 0.020 | 0.039 |
|  |  |  |  |  |  |  |  |  |  |  |  |  |  |  |  |  |  |
| Nonhalophiles | ABAC | 0.080 | 0.059 | 0.026 | 0.075 | 0.060 | 0.050 | 0.055 | 0.077 | 0.109 | 0.105 | 0.079 | 0.075 | 0.030 | 0.040 | 0.023 | 0.057 |
|  | APER | 0.061 | 0.042 | 0.094 | 0.076 | 0.032 | 0.051 | 0.014 | 0.101 | 0.120 | 0.097 | 0.088 | 0.093 | 0.038 | 0.032 | 0.021 | 0.040 |
|  | AZOA | 0.055 | 0.049 | 0.023 | 0.068 | 0.057 | 0.053 | 0.076 | 0.097 | 0.114 | 0.124 | 0.082 | 0.076 | 0.023 | 0.032 | 0.027 | 0.044 |
|  | BLON | 0.074 | 0.062 | 0.019 | 0.078 | 0.056 | 0.047 | 0.054 | 0.073 | 0.122 | 0.111 | 0.080 | 0.077 | 0.028 | 0.046 | 0.024 | 0.048 |
|  | CCRE | 0.059 | 0.052 | 0.021 | 0.065 | 0.050 | 0.055 | 0.070 | 0.093 | 0.112 | 0.137 | 0.090 | 0.076 | 0.023 | 0.033 | 0.023 | 0.043 |
|  | ECOL | 0.083 | 0.054 | 0.028 | 0.087 | 0.067 | 0.044 | 0.052 | 0.079 | 0.109 | 0.095 | 0.074 | 0.071 | 0.030 | 0.033 | 0.028 | 0.066 |
|  | GVIO | 0.056 | 0.052 | 0.029 | 0.060 | 0.060 | 0.056 | 0.069 | 0.093 | 0.110 | 0.112 | 0.083 | 0.076 | 0.028 | 0.031 | 0.026 | 0.058 |
|  | MTHA | 0.079 | 0.049 | 0.072 | 0.106 | 0.038 | 0.043 | 0.014 | 0.088 | 0.140 | 0.073 | 0.079 | 0.077 | 0.034 | 0.042 | 0.022 | 0.043 |
|  | MTHP | 0.069 | 0.046 | 0.077 | 0.103 | 0.039 | 0.045 | 0.022 | 0.089 | 0.131 | 0.083 | 0.079 | 0.078 | 0.032 | 0.043 | 0.026 | 0.039 |
|  | PCAL | 0.073 | 0.043 | 0.060 | 0.066 | 0.037 | 0.052 | 0.023 | 0.081 | 0.113 | 0.108 | 0.079 | 0.106 | 0.044 | 0.031 | 0.024 | 0.062 |
|  | PLUT | 0.073 | 0.053 | 0.037 | 0.087 | 0.051 | 0.046 | 0.051 | 0.097 | 0.119 | 0.096 | 0.081 | 0.069 | 0.029 | 0.043 | 0.022 | 0.046 |
|  | POLA | 0.065 | 0.052 | 0.029 | 0.071 | 0.063 | 0.052 | 0.060 | 0.092 | 0.102 | 0.123 | 0.082 | 0.076 | 0.024 | 0.033 | 0.026 | 0.050 |
|  | PPRO | 0.074 | 0.052 | 0.038 | 0.085 | 0.055 | 0.046 | 0.055 | 0.095 | 0.117 | 0.093 | 0.079 | 0.069 | 0.028 | 0.039 | 0.026 | 0.050 |
|  | PPUT | 0.063 | 0.048 | 0.032 | 0.068 | 0.070 | 0.049 | 0.063 | 0.100 | 0.109 | 0.112 | 0.081 | 0.073 | 0.026 | 0.027 | 0.027 | 0.053 |
|  | PTHE | 0.089 | 0.045 | 0.046 | 0.084 | 0.047 | 0.046 | 0.039 | 0.081 | 0.118 | 0.095 | 0.085 | 0.078 | 0.033 | 0.030 | 0.023 | 0.061 |
|  | RCAS | 0.044 | 0.056 | 0.024 | 0.077 | 0.057 | 0.059 | 0.078 | 0.089 | 0.109 | 0.120 | 0.077 | 0.076 | 0.028 | 0.028 | 0.026 | 0.052 |
|  | SBOY | 0.082 | 0.053 | 0.029 | 0.085 | 0.068 | 0.044 | 0.055 | 0.082 | 0.109 | 0.094 | 0.073 | 0.071 | 0.031 | 0.033 | 0.028 | 0.063 |
|  | SYNE | 0.051 | 0.049 | 0.038 | 0.062 | 0.068 | 0.055 | 0.065 | 0.105 | 0.110 | 0.106 | 0.083 | 0.071 | 0.019 | 0.034 | 0.032 | 0.052 |
|  | TACI | 0.099 | 0.048 | 0.062 | 0.121 | 0.038 | 0.039 | 0.013 | 0.073 | 0.117 | 0.069 | 0.072 | 0.072 | 0.047 | 0.054 | 0.016 | 0.057 |
|  | TKOD | 0.100 | 0.046 | 0.066 | 0.092 | 0.034 | 0.044 | 0.014 | 0.095 | 0.135 | 0.074 | 0.076 | 0.083 | 0.039 | 0.028 | 0.021 | 0.053 |
|  | TMAR | 0.112 | 0.045 | 0.062 | 0.095 | 0.036 | 0.040 | 0.010 | 0.084 | 0.139 | 0.058 | 0.069 | 0.087 | 0.037 | 0.040 | 0.020 | 0.068 |
|  | TPEN | 0.072 | 0.041 | 0.076 | 0.062 | 0.032 | 0.047 | 0.020 | 0.102 | 0.119 | 0.093 | 0.079 | 0.103 | 0.041 | 0.039 | 0.021 | 0.052 |
|  | UMET | 0.090 | 0.056 | 0.047 | 0.100 | 0.045 | 0.045 | 0.030 | 0.082 | 0.117 | 0.087 | 0.078 | 0.076 | 0.038 | 0.039 | 0.024 | 0.047 |
|  | YPES | 0.084 | 0.054 | 0.032 | 0.088 | 0.072 | 0.044 | 0.049 | 0.063 | 0.106 | 0.092 | 0.072 | 0.068 | 0.032 | 0.035 | 0.025 | 0.084 |

+ or * indicates significant (p < 10^-2^) overrepresentation of corresponding dinucleotide frequencies for halophiles or non-halophiles dataset

respectively by Kolmogorov-Smirnov two-sample test.
