# Supplementary material for: Molecular signature of hypersaline adaptation: insights from genome and proteome composition of halophilic prokaryotes
Source: Genome Biol. 2008 Apr 9;9(4):R70. doi: 10.1186/gb-2008-9-4-r70 (PMC2643941; doi:10.1186/gb-2008-9-4-r70)
Supplement: Additional data file 12 — RSCU of genes at the positive and negative extremes (5,000 each) of axis 3 of COA on RSCU. [file gb-2008-9-4-r70-S12.doc]

**Additional Data File 12: Relative Synonymous Codon Usage of positive and negative extreme genes (5000 each) of axis 3 of COA on RSCU**

| Amino  Acid | Codon | Positive extreme of axis 3 | Negative extreme of axis 3 | Amino  Acid | Codon | Positive extreme of axis 3 | Negative extreme of axis 3 |
| --- | --- | --- | --- | --- | --- | --- | --- |
|  |  | RSCU | RSCU |  |  | RSCU | RSCU |
| Phe | UUU | 0.58 | 0.66 | Tyr | UAU | 0.71 | 0.65 |
|  | UUC | 1.42 | 1.34 |  | UAC | 1.29 | 1.35 |
| Leu | UUA | 0.25 | 0.22 | ter | UAA | 0.84 | 1.37 |
|  | UUG | 0.42 | 0.55 | ter | UAG | 0.79 | 0.38 |
|  | CUU | 1.11* | 0.40 | His | CAU | 0.64 | 0.68 |
|  | CUC | 2.55* | 0.67 |  | CAC | 1.36 | 1.32 |
|  | CUA | 0.27 | 0.13 | Gln | CAA | 0.60 | 0.41 |
|  | CUG | 1.40 | 4.03* |  | CAG | 1.40 | 1.59 |
| Ile | AUU | 0.98 | 0.91 | Asn | AAU | 0.73* | 0.45 |
|  | AUC | 1.74 | 1.79 |  | AAC | 1.27 | 1.55* |
|  | AUA | 0.28 | 0.30 | Lys | AAA | 0.88 | 0.95 |
| Met | AUG | 1.00 | 1.00 |  | AAG | 1.12 | 1.05 |
| Val | GUU | 0.82 | 0.80 | Asp | GAU | 0.70 | 0.74 |
|  | GUC | 2.04* | 0.93 |  | GAC | 1.30 | 1.26 |
|  | GUA | 0.27 | 0.50* | Glu | GAA | 0.80 | 1.11* |
|  | GUG | 0.88 | 1.77* |  | GAG | 1.20* | 0.89 |
| Ser | UCU | 0.47 | 0.69 | Cys | UGU | 1.49* | 0.26 |
|  | UCC | 0.84 | 1.41* |  | UGC | 0.51 | 1.74* |
|  | UCA | 1.06* | 0.29 | ter | UGA | 1.37 | 1.25 |
|  | UCG | 1.78* | 0.91 | Trp | UGG | 1.00 | 1.00 |
| Pro | CCU | 0.36 | 0.51 | Arg | CGU | 0.81 | 1.73* |
|  | CCC | 0.85 | 0.89 |  | CGC | 1.52 | 2.80* |
|  | CCA | 0.96* | 0.45 |  | CGA | 1.38* | 0.06 |
|  | CCG | 1.82 | 2.15 |  | CGG | 1.96* | 0.45 |
| Thr | ACU | 0.51 | 0.54 | Ser | AGU | 0.70* | 0.39 |
|  | ACC | 1.13 | 2.59* |  | AGC | 1.15 | 2.31* |
|  | ACA | 0.92* | 0.23 | Arg | AGA | 0.23 | 0.16 |
|  | ACG | 1.44* | 0.63 |  | AGG | 0.11 | 0.80 |
| Ala | GCU | 0.59 | 0.57 | Gly | GGU | 0.78 | 1.04* |
|  | GCC | 1.27 | 1.88* |  | GGC | 1.55 | 2.30* |
|  | GCA | 0.84* | 0.55 |  | GGA | 0.73* | 0.21 |
|  | GCG | 1.30* | 0.99 |  | GGG | 0.93* | 0.46 |

**Note:** * indicates corresponding codons are significantly over-expressed among the positive extreme (halophiles) or negative extreme (nonhalophiles) genes (p<10^-4^). For statistical tests we randomly chosen 500 genes from both end for 20 times and calculated the codon count and RSCU. The significant (p<10^-4^) trends for the whole dataset are also consistent with sets taken randomly for 20 times.
